# Effects of Transcranial Direct Current Stimulation on Speech and Voice Changes in Parkinson's Disease: a Systematic Review with Meta-Analysis

**DOI:** 10.1590/2317-1782/e20250002en

**Published:** 2026-02-27

**Authors:** Renata Serrano de Andrade Pinheiro, Hercílio Barbosa da Silva, Bruno Braga Guimarães, Ivonaldo Leidson Barbosa Lima, Rafael Nóbrega Bandeira, Nelson Torro Alves

**Affiliations:** 1 Programa de Pós-graduação em Neurociências Cognitiva e Comportamento – PpgNeC, Departamento de Psicologia, Universidade Federal da Paraíba – UFPB - João Pessoa (PB), Brasil.; 2 Departamento de Pesquisa do Neuronus, Instituto Transdisciplinar de Ensino e Pesquisa sobre o Cérebro - Goiânia (GO), Brasil.; 3 Departamento de Fonoaudiologia, Universidade Federal do Rio Grande do Norte – UFRN, Natal (RN), Brasil.; 4 Departamento de Fonoaudiologia, Universidade Federal da Paraíba – UFPB - João Pessoa (PB), Brasil.

**Keywords:** Parkinson's disease, Transcranial Direct Current Stimulation, Voice, Voice Quality, Speech

## Abstract

**Purpose:**

This meta-analysis aims to evaluate the existing evidence on the effects of tDCS on speech and voice alterations in patients with PD.

**Search strategies:**

PubMed, LILACS, EMBASE, Cochrane Central Register of Controlled Trials, Science Direct, Web of Science, Scopus, and gray literature searches: Google Scholar and Open Grey. The search included the descriptors: "Parkinson Disease, Transcranial Direct Current Stimulation, Voice, Speech" combined with AND and OR.

**Selection criteria:**

Patients with Parkinson's Disease, both sexes. Use of Transcranial Direct Current Stimulation (tDCS) to treat voice and speech parameters in patients with Parkinson's Disease.

**Data analysis:**

A total of 1,345 articles were included, 14 articles in the systematic review and 6 articles in the meta-analysis. The risk of bias and level of evidence were assessed using REVIEW MANAGER 5.4.1 and GRADE software.

**Results:**

The results showed an overall effect size of tDCS of Z=0.89 (P=0.37). Studies targeting the prefrontal cortex (PFC) showed a larger effect size of 1.38 (P=0.17), thus demonstrating a greater impact on speech and voice outcomes with the use of tDCS for PD. Three studies presented a low risk of bias, and three studies presented an unclear risk.

**Conclusion:**

Despite the small number of studies, the findings of this meta-analysis suggest the potential applicability of tDCS as an adjunctive tool in the treatment of voice and speech disorders in patients with PD.

## INTRODUCTION 

Increasing life expectancy and advances in the diagnosis and treatment of degenerative diseases, which primarily occur in people over 65, have highlighted Parkinson's Disease (PD) as one of the most prevalent diseases today^(1)^. PD is characterized by the degeneration of neurons in the substantia nigra of the midbrain, which causes a decrease in the production of the neurotransmitter dopamine. The pathophysiology of classic PD signs includes dysfunction in the basal ganglia circuits as a determining factor in the emergence of changes in movement control, resulting in motor and non-motor symptoms, with the presence of voice and speech dysfunctions, characterized by dysarthrophonia and/or dysarthria^(2)^.

Feedforward motor and sensory feedback mechanisms comprise current models of speech and propose the complex role of the brain in performing this mechanism. The detrimental effects on voice motor control mechanisms in PD occur due to the degeneration of dopaminergic neurons, resulting in impairments in movement mechanisms that lead to speech motor disorders: voice tremor, reduced voice volume, and difficulties with intonation^(3)^. Changes in voice and speech motor control in PD are evidenced by decreased excitatory input to the cortex, which causes and gives rise to physiological deficits in the peripheral systems (laryngeal, articulatory, and respiratory), as well as decreased cortical activity in the speech production network^(4)^.

In recent years, a growing number of researchers have dedicated themselves to studying the effects of non-invasive brain stimulation techniques, such as Transcranial Direct Current Stimulation (tDCS), in managing the difficulties caused by PD. tDCS is a technique in which a low-intensity electrical current is administered through two surface electrodes—an anode and a cathode—positioned on the scalp^(5)^. This current can induce changes in the resting potential of the neuronal membrane, modulating its excitability and making the brain region below the electrodes more or less active, depending on the polarity of the current. Repeatedly applying this stimulation to a specific target region of the brain can modulate the underlying neural networks through the mechanism of postsynaptic neuroplasticity, producing significant functional or behavioral changes^(5)^.

Despite the growing number of studies using tDCS to treat motor and non-motor functions in individuals with PD, there are still gaps and questions to be answered regarding the effectiveness of this therapeutic resource in this population. To our knowledge, there are still no studies on the effects of tDCS on voice and speech changes caused by PD. Therefore, we believe it is important to conduct a Systematic Review of the scientific bases and verify, through a Meta-Analysis, how tDCS Neuromodulation is bringing benefits and enhancing the therapeutic results of Speech-Language Pathology (SLP) on the voice and speech of individuals with PD.

The objective of this study was to perform a systematic review with meta-analysis of articles that use Transcranial Direct Current Stimulation as an adjuvant technological resource for the treatment of speech and voice in individuals with Parkinson's Disease.

## METHOD

This Systematic Review (SR) protocol was registered with the International Prospective Register of Systematic Reviews (PRÓSPERO) platform prior to its implementation, under number CRD42024542291, after a search and verification of the absence of SR studies with meta-analysis addressing this topic and evaluation.

### Study design

This protocol included the following aspects related to the research topic: the research question, the criteria for selecting and searching for articles in the databases explored, eligibility, data extraction, data analysis, and the manner in which outcomes were assessed. There were no restrictions on language or year of publication for the articles included in this SR. The acronym PICOS (Patient, Intervention, Comparator, Outcomes and Study design) was used for the guiding research question of this systematic review according to the PRISMA protocol: “What are the effects and evidence on the voice and speech of individuals affected by Parkinson's Disease who used transcranial direct current stimulation-tDCS as a therapeutic resource?”.

The articles included in this SR were limited by language (Portuguese, English, or Spanish) and publication year within the last ten years. They were selected according to the inclusion and exclusion criteria. Data collection for this SR in the databases was conducted from May 1, 2024, to July 31, 2024.

The criteria used by the acronym PICOS for this SR are described in Table 1 below. The table also addresses the inclusion and exclusion criteria for the studies in this SR.

**Table 1:** PICOS criteria for selecting studies for this Systematic Review.

	INCLUSION	EXCLUSION
**PATIENT**	Patients with Parkinson's disease, both sexes, in stages 2 or 3 of the Hoehn-Yahr PD scale.	- Individual health condition: unstable hemodynamics, other neurological disease, or untreated psychiatric illness.
**(Population)**		- Use of DBS or metal in the brain, or any reason that prevents tDCS.
		- Hearing impairment or use of hearing aids or cochlear implants.
**INTERVENTION**	Use of Transcranial Direct Current Stimulation (tDCS) to treat voice and speech parameters in patients with Parkinson's disease.	Studies that do not use tDCS in the proposed treatment.
**(Intervention)**	(a-tDCS, c-tDCS, s-tDCS, or sham)	
	
**COMPARISON**	Areas of application of the 10-20 System Protocol for tDCS, focusing on voice and speech for individuals with Parkinson's disease.	Studies that do not identify the area stimulated with tDCS.
**(Comparison)**		

**OUTCOME**	Improvement in voice production and acoustic parameters, speech fluency and intelligibility, and reduction in signs and symptoms of voice and speech disorders.	Studies that do not have voice and speech outcomes.
**(Outcomes)**		

**STUDY DESIGN**	Intervention studies (cross-sectional or longitudinal), with randomized or non-randomized clinical trials, and other studies addressing Transcranial Direct Current Stimulation (tDCS) in individuals with PD.	Accuracy, Prevalence, Prognosis.
**(Study Design)**		

**DATA REPORT**	Use data to analyze and estimate the effects of tDCS associated with voice and speech training or assessment in PD.	
**(Data Report)**		

**TYPE OF PUBLICATIONS**	Must have been published in a peer-reviewed journal within the last ten years and in any language.	
**(Type of Publications)**		


**Caption:** tDCS - Transcranial direct current stimulation atDCS - Anodal Transcranial Direct Current Stimulation ctDCS - Cathodal Transcranial Direct Current Stimulation sham or stDCS - Simulated Transcranial Direct Current Stimulation DP - Parkinson's Disease DBS - Deep Brain Stimulation AASI - Individual Sound Amplification Device.

### Literature search and selection criteria

Initially, a search and identification of terms and descriptors to be used in the Health Sciences Descriptors (DeCS) and Medical Subject Heading (MeSH) platform were performed for the research topic (Parkinson's), the therapeutic resource (Transcranial Direct Current Stimulation - tDCS), and the expected outcomes of the studies included in this SR for speech and voice in individuals with Parkinson's undergoing this therapeutic activity.

When planning the search strategy, the terms and descriptors were found and used to conduct the systematic review. The descriptors used in this review were: "Parkinson's Disease," "Transcranial Direct Current Stimulation," "Voice," and "Speech," and their Portuguese equivalents, "Doença de Parkinson," "Estimulação Transcraniana por Corrente Contínua," "Voz," and "Fala." The AND and OR combinations were used according to the combinations between the descriptors in the databases searched.

Searches for terms and descriptors were performed in the following databases: PubMed, Cochrane Central Register of Controlled Trials, Embase, Scopus, Web of Science, Scielo, Lilacs, Science Direct, and Medline. The gray literature databases Google Scholar and Open Grey also participated in the research.

#### Data collection and analysis

The Preferred Reporting Items for Systematic Reviews and Meta-Analyses (PRISMA-2020)^(6)^ protocol was used for this systematic review. This protocol is recommended because it provides greater accuracy in describing and reporting systematic reviews.

After completing the database search, the SR articles were exported to the ENdNote® reference manager (EndNote/Clarivate Analytics, PA, USA) for organization and removal of duplicate articles. The articles were then exported to Rayyan® software (Qatar Computing Research Institute, Doha, Qatar), where studies were blindly and independently selected by two reviewers. Decisions were recorded on the platform and disagreements were resolved by consensus. Articles were selected based on the eligibility criteria and the inclusion and exclusion criteria. After completing the data collection, analysis, interpretation, and discussion of the results, PRISMA^(6)^ was followed to prepare the final report of this Systematic Review on the Effects and Evidence of Transcranial Direct Current Stimulation on Voice and Speech Changes in Individuals with PD. The meta-analysis followed the random-effects model for the statistical models applied through the REVIEW MANAGER 5.4.1 software. Subgroup analysis was performed to assess the robustness of the results, observed in the forest plot graphs.

#### Quality Assessment

The methodological quality of the research articles was assessed following the guidelines that aid in the development of a high-quality SR: The Cochrane Reviewers' Handbook^(7)^. The RoB 2.0 tool^(8)^ (Revised Cochrane risk-of-bias tool for randomized trials) recommended by Cochrane to assess the risk of bias in randomized clinical trials will be used to assess the risk of bias, observing the following domains: bias in the randomization process, deviations from the intended intervention due to missing data, in the measurement of outcomes, and in the reporting of outcomes^(7)^. To assess the effect size, the REVIEW MANAGER 5.4.1 software was used, which displays the forest plot with the effect size of the studies, the degree of confidence, and the heterogeneity that the study results may present. The precision of the effect sizes was estimated using a 95% confidence interval.

## RESULTS

### Data extraction

The PRISMA^(5)^ diagram describes the steps of the Systematic Review. It shows the results through the following topics: ***Identification*** of 1,345 articles found in different databases: PubMed, Cochrane Central Register of Controlled Trials, Embase, Scopus, Web of Science, Scielo, Lilacs, Science Direct, Medline, and the gray literature databases Google Scholar and Open Grey. In the ***Screening*** section, the 1.345 articles were exported to Rayann for selection and screening by reading the titles and abstracts. Eleven duplicate articles were removed from the total search results in EndNote after automatic selection in Rayyan. A total of 1.279 articles were excluded for not being relevant to the research question, leaving a total of 66 articles for full reading, following the eligibility criteria for the database search proposed in the PRÓSPERO Systematic Review Protocol. In the end, 14 articles were analyzed for selection and analysis of results described in Tables 2 and 3, with a meta-analysis was performed using the PRISMA (2020) ***Included*** section for this SR, articles with sources (n=1), results (n=2), or comparators (n=5) that did not meet the meta-analysis requirements were excluded. Some studies presented a different population, did not use Transcranial Direct Current Stimulation (tDCS) as a technological resource, or presented another type of outcome in the treatment intervention other than voice or speech. At the end of the analysis and exclusions, 6 articles were included for the Systematic Review with Meta-analysis.

**Table 2:** Description of Theoretical or Conceptual and Systematic Review articles with tDCS for voice and/or speech treatment in PD.

**AUTHORS/YEAR**	**JOURNAL/** **PUBLISHMENT DOI**	**TYPE OF STUDY/TREATMENT TECHNIQUE**	**OBJECTIVE**	**tDCS METHOD/PARAMETERS**	**MAIN FINDINGS**	**CONCLUSION**
FOX, MD; BUCKNER, RL et al (2014)	PNAS PLUS **DOI:** 10.1073/pnas.1405003111	**A. CONCEPTUAL OR THEORETICAL** Invasive and Noninvasive Brain Stimulation in Psychiatric and Neurological Disorders	Identify diseases treated with invasive and non-invasive stimulation, list the stimulation sites considered most effective in each disease using resting-state functional connectivity MRI.	- The literature search revealed 14 different psychiatric or neurological diseases with published reports of efficacy for both invasive brain stimulation and non-invasive brain stimulation. - Correlations between DBS regions and all other brain voxels were computed and related to sites with evidence of efficacy as targets for non-invasive brain stimulation (TMS or tDCS).	- **Diseases and stimulation areas:** **Alzheimer's disease** - DBS (fornix), TMS or tDCS (bilateral DLPFC + parietal/temporal), **Depression** - DBS (subgenual, VC/VS, NA, MFB, habenula), TMS or tDCS (e-DLPFC, d-DLPFC), **Epilepsy** - DBS (thalamus (AN, CM), MTL), TMS or tDCS (EEG active focus, cerebellum), **Pain** - DBS (PAG, thalamus (VPL/VPM)), TMS or tDCS (M1), **Parkinson's disease** - DBS (STN, GPi), TMS or tDCS (M1, SMA), and others.	This work supports a network perspective for understanding and treating neuropsychiatric diseases, highlighting the therapeutic potential of the brain as a target for network modulation.
MITERKO, LN ; BAKER, KB et al (2019)	The Cerebellum **DOI:** 10.1007/s12311-019-01041-5	Consensus Article: Experimental Neurostimulation of the Cerebellum	Report the most advanced techniques for manipulating cerebellar circuits in humans and animal models and define the main obstacles and issues for moving forward.	-Recent paradigms of animal and human stimulation that utilized the cerebellum were presented, identifying key successes and failures, which are essential for improvements in human therapy. Important obstacles were outlined and possible ways to overcome them were suggested.	- Recently described anatomical connections between the cerebellum and basal ganglia expand the potential applications of cerebellar tDCS in dystonias and other disorders. - Cerebellar tDCS modifies cerebellar cortex excitability with minor side effects. - Cerebellar anodal tDCS improves the kinematics of handwriting and circle-drawing tasks in patients with writing dystonia and promotes the rehabilitation of speech and language deficits.	- Anodal tDCS increased the ability to learn from mistakes, as well as form and retain motor memory. The cerebellum also has a broad influence and contributes significantly to non-motor domains, such as cognition. - Positive results of anodal tDCS on verbal fluency and pain perception in healthy individuals and cognitive symptoms in Parkinson's patients.
						
MANTO,M, et al (2022)	The Cerebellum **DOI:** 10.1007/s12311-021-01344-6	New Directions and Next Steps in the Non-Invasive Approach to Cerebellar Brain Stimulation in Health and Disease	To report advances made in the use of cerebellar NIBS and reach consensus on future steps to move forward.	- Use of TMS to explore cerebellar neurophysiology; current knowledge on cerebello- cerebellar tDCS; the role of animal models in cerebellar applications of NIBS; clinical application of cerebellar NIBS (motor learning, stroke recovery, speech and language functions, neuropsychiatric and movement disorders, and pain syndromes).	**-** Functional neuroimaging has shown a cerebellar role in verbal fluency in neurostimulation research. Anodal cerebellar tDCS (right posterolateral cerebellum) improved phonemic fluency. Lin et al. - Nineteen patients with SCA underwent neuronavigated cTBS (right cerebellum vs. sham stimulation) producing sustained vowels while perceiving the pitch change in their voice. Compared to sham, cerebellar cTBS led to smaller magnitudes of vocal compensations for pitch disturbances, demonstrating that cerebellar NIBS can modulate abnormal auditory-vocal integration in SCA.	Non-invasive cerebellar stimulation represents a promising tool for therapeutic purposes, both in motor, cognitive, and psychiatric pathological conditions. Targeting tDCS to the cerebellum to indirectly affect cortical and subcortical activity may be effective in alleviating symptoms of various pathologies, as well as improving cognitive functions or motor learning in healthy individuals.
MONTEMURRO,NALIAGA,N et al (2022)	International Journal of Environmental Research and Public Health **DOI:** 10.3390/ijerph19148799	New targets and new technologies in the treatment of Parkinson's disease	The aim of this narrative review is to show new targets in DBS surgery, as well as new technologies that are under study and have shown promising results to date.	Literature review of databases for an overview of new therapeutic targets for PD, as well as their limitations. Review research that will become effective and routine treatments for PD in the near future.	- Non-invasive modulation of spontaneous neuronal activity with electric current (1–2 mA) by two or more electrodes on the scalp for the regulation of neuronal membrane potentials, changes in neurotransmitters, glia and microvessels. - Provides neuronal plasticity by generating long-term synapses (BDNF) - (tDCS) affects the motor symptoms of the disease, with the most prominent results being related to rehabilitation.	Progress in device development is reflected in increasingly personalized treatments and the targeting of new targets. These new devices offer the advantage of being lower cost and minimally invasive or non-invasive.
SALUJA, A; GOYAL,V; DHAMIJA, RK (2022)	Indian Academy of Neurology - Neuro‑Rehabilitation Supplement **DOI:** 10.4103/aian.aian_164_22	Multimodal rehabilitation therapy in Parkinson's disease and related disorders	To evaluate and summarize current evidence on rehabilitation strategies in PD and atypical parkinsonian syndromes. Highlight evidence and advances in the rehabilitation of patients with parkinsonism.	-A literature search was conducted in MEDLINE (PubMed) and Cochrane. Relevant results from the full text and abstracts of the studies were reviewed. Randomized clinical trials (RCTs), trial protocols, consensus guidelines, cohort studies, systematic reviews, meta-analyses, review articles, pilot studies, case reports, observational studies, and pre-post studies.	- Speech and Language Rehabilitation in PD - 89% of PD patients may develop speech and voice disorders (reduced volume, monotonous voice, and breathy quality), articulation (imprecise consonants and vowel centralization), and frequency (increased, decreased, and variable). Speech-language therapy improved sound pressure levels during sustained phonation, reduced Voice Handicap Index scores, and improved reading of the Rainbow Passage and monologue.	Incorporating rehabilitation strategies, particularly early in the disease course, can improve motor and non-motor symptoms, along with the Quality of Life of patients with PD.
WEISMER, G et al (2023)	Brain Sciences **DOI:** 10.3390/brainsci13050768	Nonverbal oromotor performance and speech motor control: theory and review of empirical evidence	Outline general issues related to non-speech oromotor tasks and their relationship to speech production	- Twelve studies were found on the relationships between nonverbal oromotor performance and speech production measures in healthy individuals (3 with positive results; 2 with mixed results; 7 with negative results). **-** 19 studies on the relationships between nonverbal oromotor performance and speech production measures in neurologically based speech disorders (3 with positive results, 5 with mixed results, and 11 with negative results).	Transcranial direct current stimulation (tDCS) was applied to jaw movements associated with speech, maximum syllable repetition, and chewing. The relationship between nonverbal oromotor performance and speech production measures was negative. The results showed that the effects of tDCS are task-dependent.	To advance the research and clinical disciplines of speech motor control, the study of speech production and its goal of providing listeners with linguistically relevant, time-varying acoustic signals is necessary and (I believe) sufficient.
BAIG, FN (2023)	Brain Stimulation DOI:10.1016/j.brs.2023.01.354	**B. SYSTEMATIC REVIEW** RS: Update on the therapeutic efficacy of non-invasive brain stimulation for dysarthria recovery: a systemic review	To collect evidence of the usefulness of NIBS in the treatment of dysarthria through the systematic evaluation of original research articles.	**DATABASES:** Google Scholar, Embase, Scopus, PubMed, Cochrane, and Web of Science. **KEYWORDS:** Dysarthria, tDCS, TMS, neurological disorder	Fourteen of the 9.244 studies were included. Nine used TMS and five evaluated tDCS. Adjunctive speech-language therapy (SLP) to NIBS was offered in six studies. Dysarthria resulted from cerebral palsy, stroke, PD, cerebellar ataxia, traumatic brain injury, and supranuclear palsy. 83% of the studies found positive effects of NIBS. The significant post-treatment difference between the sham and treatment groups was associated only with the sequential movement rate (Hedges' g = 0.79, 95% CI [0.075, 1.50] P = 0.03), favoring tDCS.	Most studies that have identified the benefits of SBNI have not provided evidence for complete recovery from dysarthria. Large-scale clinical investigations are needed to evaluate the effectiveness of SBNI in treating dysarthria.
BALZANA, P et al (2022)	Annals of Physical and Rehabilitation Medicine DOI: 10.1016/j.rehab.2021.101580	RS: Non-invasive brain stimulation for the treatment of neurogenic dysarthria.	To examine the evidence base for non-invasive central and peripheral stimulation to improve speech-related functions in people with dysarthria and determine the effects of an intervention.	**DATABASES:** **MEDLINE** PsychINFO EMBASE CINAHL Linguistics and Language Behavioral Abstracts Web of Science The Cochrane Register of Controlled Trials and 2 trial registries were completed. **KEYWORDS:** Brain and electrical stimulation, Dysarthria, and Research design.	A total of 6,186 studies were identified; 10 studies (6 randomized controlled trials and 4 crossover studies) met the inclusion criteria. The 10 studies (268 adults with Parkinson's disease, stroke, and neurodegenerative cerebellar ataxia) focused on NIBS (6 TMS; 3 tDCS; and 1 tACS). Most trials reported one or more positive effects of stimulation on dysarthria-related features. All 3 studies provided anodal stimulation over the cerebellum or M1, with a current intensity of 2 mA. tDCS was administered for 20 (n=2) or 30 (n=1) min. In all trials, stimulation was administered 5 times per week for a total of 10 sessions.	The evidence for the use of noninvasive brain stimulation in the treatment of dysarthria remains inconclusive. Research trials that provide reliable and replicable results are needed.

**Caption:** tDCS - Transcranial direct current stimulation; atDCS - Anodal Transcranial Direct Current Stimulation; ctDCS - Cathodal Transcranial Direct Current Stimulation; sham or stDCS - Simulated Transcranial Direct Current Stimulation; PD - Parkinson's disease; AVC - Stroke; DBS - Deep Brain Stimulation; VAS - Visual Analog Scale; NHR - Harmonic-to-Noise Ratio; SMA - Supplemental Motor Area; UPDRS - Unified Parkinson's Disease Rating Scale; DLPFC - Dorsolateral Prefrontal Cortex; TPC - Left Temporoparietal Cortex; FP2 - Orbitofrontal Cortex; LSVT - Lee Silverman Voice Treatment; MoCA - Montreal Cognitive Assessment

**Table 3:** Description of empirical articles included in the Meta-Analysis on Transcranial Direct Current Stimulation (tDCS) for the treatment of voice or speech in PD.

**AUTHORS/ YEAR**	**JOURNAL /PUBLISHMENT DOI**	**TYPE OF STUDY / TREATMENT TECHNIQUE**	**OBJECTIVE**	**tDCS METHOD / PARAMETERS**	**MAIN FINDINGS**	**CONCLUSION**
PEREIRA, JB et al (2013)	Brain Stimulation **DOI:** 10.1016/j.brs.2012.01.006	**C. EMPIRICAL** **Clinical Trial** **tDCS** Modulation of verbal fluency networks by transcranial direct current stimulation (tDCS) in Parkinson's disease.	To evaluate the effects of tDCS on the functional networks of phonemic and semantic fluency in patients with PD.	- 16 patients with idiopathic PD; N=16 - Unified Parkinson's Disease Rating Scale (UPDRS) and Hoehn & Yahr Scale; Mini-Mental and Depression Scale; - Neuropsychological tests. tDCS at 2 mA for 20 min; atDCS over F3 (left DLPFC) or P3-T5 (left TPC); and ctDCS over right FP2.	- Effects of tDCS on verbal fluency (F(1,15) = 14.079, P<0.002), DLPFC stimulation increased the number of words produced. - Effects of tDCS on semantic fluency - F(1,15) = 3.092, P<0.102) DLPFC stimulation increased the number of words produced.	tDCS improved functional connectivity in verbal fluency and deactivation task-related networks more when applied over the DLPFC than did TPC in PD.
PIETROBON et al. (2021)	Audiology Communication Research **DOI:** 10.1590/2317-6431-2020-2343	Clinical Case tDCS Audiovisual production therapy combined with transcranial current stimulation improves naming in a patient with Broca's aphasia and Parkinson's disease	Stimulating Broca's area with tDCS due to the deficit in naming tasks, in the left inferior prefrontal cortex (Broca's area).	-A 79-year-old patient with PD (stage 4 on the Hoehn and Yahr scale) and chronic nonfluent post-stroke aphasia, with severe naming deficits; N=1 - Combination of audiovisual production therapy and anodal tDCS (2 mA) applied to the left inferior prefrontal cortex (F7), nine 20-minute sessions + naming of images of common objects with the aid of short videos.	Significant increase in naming scores between pre- and post-treatment, both for trained and untrained but phonemically similar images (generalization).	The results presented initial evidence that audiovisual production therapy combined with anodal tDCS over Broca's area may represent a viable alternative for patients with severe naming deficits.
LEYDON,C et al. (2022)	Perspectives - SIG 2 Neurogenic Communication Disorders **DOI:** 10.1044/2021_PERSP-21-00040	Clinical Trial tDCS Speech Changes After Transcranial Direct Current Stimulation in Individuals Diagnosed with Parkinson's Disease	To examine the impact of tDCS on speech production in individuals diagnosed with PD.	-Six men, aged 65 to 78, diagnosed with PD. N=6 -A double-blind, crossover, controlled study of the immediate effects of tDCS on the premotor cortex for speech production. - - Auditory-perceptual (AVS) and acoustic voice outcomes (fundamental frequency, intensity, NHR, and speech rate) pre- and post-anodal and sham simulation	-Speech rate was below normative levels for all participants before tDCS. Speech rate increased significantly in conversation after stimulation, but not in sham tDCS, exceeding normative values ​​for two participants. NHR increased after stimulation. There were no significant differences in other measures of disturbance; perceptual ratings, fundamental frequency, or intensity were recorded.	All participants completed the protocol without discomfort. The observed increase in speech rate needs to be interpreted in the context of arousal resulting from tDCS neurostimulation.
ROSA, RR, et al. (2023)	Audiology Communication Research **DOI:** 10.1590/2317-6431-2023-2795pt	Clinical Case tDCS Effects of transcranial direct current stimulation (tDCS) on voice and speech in Parkinson's disease: a case report	To compare the effectiveness of conventional speech therapy combined with tDCS and tDCS alone.	-Two male patients with PD and hypokinetic dysarthria who underwent speech-language pathology evaluation. N=2. S1 - 10 sessions of 20 minutes of tDCS S2 - 10 sessions of 20 minutes of tDCS + speech-language pathology therapy Evaluated pre- and post-operatively with a 30-day follow-up. 10 consecutive days of anodal tDCS in the primary motor cortex (M1), 10-20 system (C3), and cathodal (FP2)	-Improvements in phonation time, velar movement, and other dysarthria measures were most significant in S1. - Acoustic analysis of the glottal source showed improvements in frequency measures and jitter, shimmer, and increased noise in S2. - S1 presented better results in the auditory-perceptual assessment of speech and voice, while S2 obtained better scores in the acoustic analysis.	Conventional speech-language therapy combined with tDCS has a more significant impact on speech and voice than tDCS alone, demonstrating the potential of tDCS as a complementary treatment for patients with PD.
JOHARI K (2024)	Cochrane Library - Cochrane Central Register of Controlled Trials	Clinical Trial tDCS Speech and Voice Outcomes After HD-tDCS on the Left SMA	To investigate the immediate and short-term effects of HD-tDCS in the left supplementary motor area (SMA) on speech and voice deficits.	24 participants with PD and 24 matched controls. N=48 Anodal tDCS on Supplemental Motor Area (SMA) 2 mA / 20 minutes 5 consecutive days Conducting a randomized clinical trial protocol registered with Cochrane regarding the immediate and short-term effects of high-definition transcranial direct current stimulation (HD tDCS) on the left supplementary motor area (SMA) on speech and voice deficits.	Growing evidence supports the use of non-invasive brain stimulation techniques such as tDCS to improve motor and non-motor symptoms in PD. However, there is limited evidence for the use of tDCS in improving speech and voice disorders in PD. Furthermore, there is no established long-term effect of tDCS on speech and voice deficits in PD.	There are no results yet, the study is ongoing.
BRABENEC, L et al (2024)	Journal of Neural Transmission **DOI:** 10.1007/s00702-024-02771-5	Clinical Trial tDCS Short-Term Effects of Transcranial Direct Current Stimulation on Motor Speech in Parkinson's Disease: A Pilot Study	Develop a program for remote treatment of HD-tDCS combined with LSVT®	-14 right-handed patients with clinically established PD. N=14 - (MoCA) for dementia, MoCA>20; ON medication without dyskinesias; presence of dysarthria symptoms -Three sessions of tDCS stimulation (anodal, cathodal, and sham stimulation) of 2 mA for 20 minutes, separated by one day, with speech tasks lasting up to 10 minutes with 150 words.	Through linear mixed model (LMM) analysis, a significant effect of the stimulation condition was observed on changes in the median duration of silences longer than 50 ms (F(2,21.8)=5.1, p=0.015), inappropriate silences that negatively impact speech rhythm and fluency.	Anodal tDCS targeting the auditory feedback area in the right hemisphere can significantly improve speech motor fluency in patients with PD.

**Caption:** tDCS - Transcranial direct current stimulation; atDCS - Anodal Transcranial Direct Current Stimulation; ctDCS - Cathodal Transcranial Direct Current Stimulation; sham or stDCS - Simulated Transcranial Direct Current Stimulation; PD - Parkinson's disease; AVC - Stroke; DBS - Deep Brain Stimulation; VAS - Visual Analog Scale; NHR - Harmonic-to-Noise Ratio; SMA - Supplemental Motor Area; UPDRS - Unified Parkinson's Disease Rating Scale; DLPFC - Dorsolateral Prefrontal Cortex; TPC - Left Temporoparietal Cortex; FP2 - Orbitofrontal Cortex; LSVT - Lee Silverman Voice Treatment; MoCA - Montreal Cognitive Assessment.

**Figure 1:**
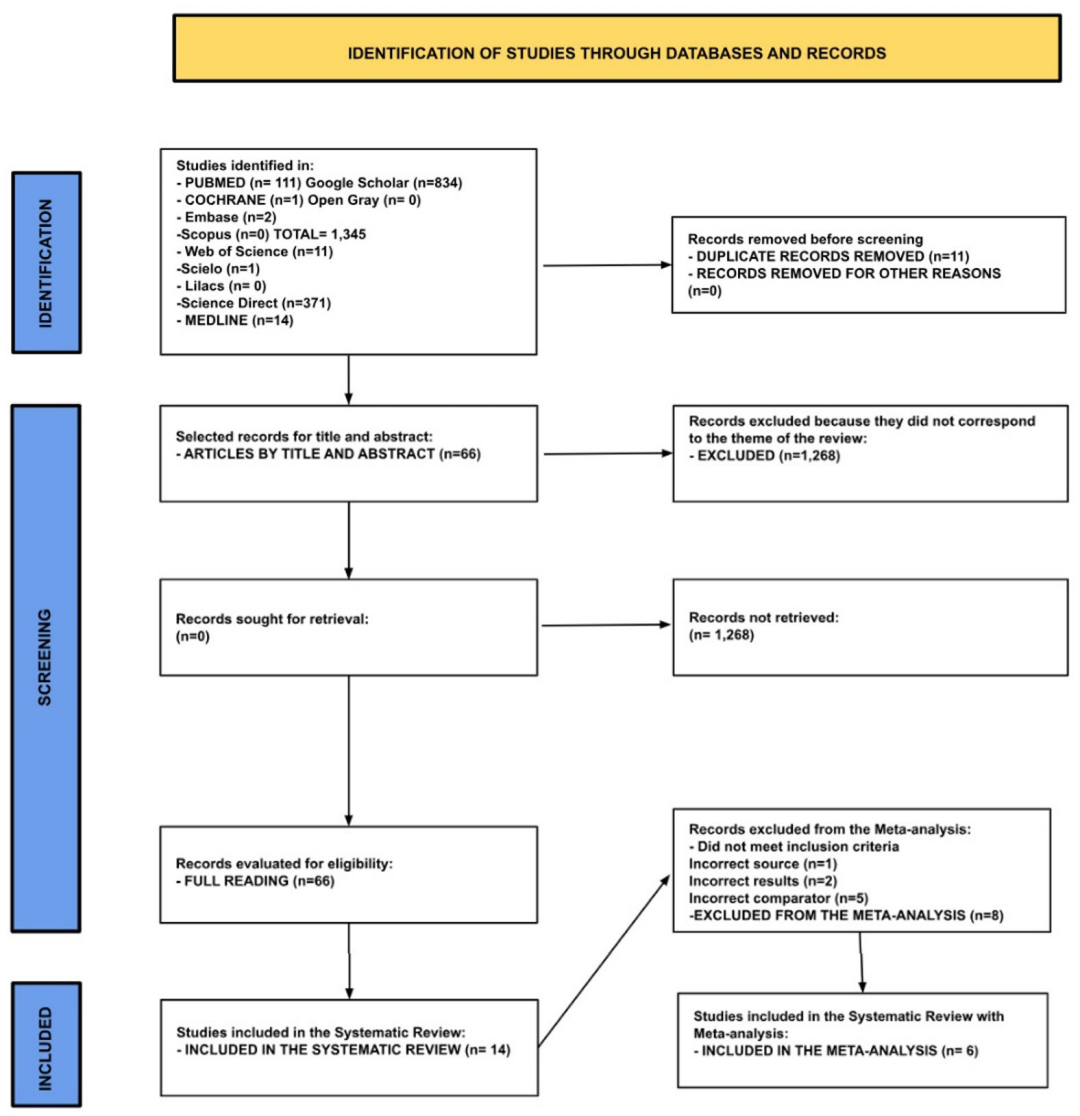
PRISMA flowchart (2020) - overview of the screening and exclusion process of the different articles.

The PRISMA(2020) Diagram with the Identification, Selection and Inclusion phases of the articles of this Systematic Review and Meta-Analysis is presented in Figure 1 below.

### Description of studies

The studies included in this Systematic Review were described by topic in tabular form and will be discussed with other studies and literature findings on the topic of the research question.

### Systematic review

Table 2 presents the eight publications selected for this review. These publications refer to theoretical, conceptual, or Systematic Review publications that lacked empirical data for inclusion in the Meta-Analysis and used Transcranial Direct Current Stimulation (tDCS) for PD. They include the authors' identification and year of publication, place of publication, DOI, objectives, method used, tDCS parameters, main findings, and the study's conclusion.

### Meta-analysis

The publications of empirical studies with the six articles that comprise this Meta-Analysis are described in Table 3, with the same topics already indicated in Table 2 for the use of tDCS for speech and voice in PD.

Table 4 lists the six empirical articles included in the meta-analysis that refer to the use of transcranial direct current stimulation (tDCS) for the treatment of Parkinson's disease with voice and speech outcomes, which will be described below. The table describes the type of neuromodulation stimulation, medication status during the session, stimulation sites (anode/cathode/sham), electrode size, current intensity (mA), stimulation duration in minutes, and number of sessions.

**Table 4:** tDCS parameters of the articles included in the Systematic Review with Meta-Analysis of tDCS for the treatment of voice and/or speech in PD.

**AUTHOR/YEAR** **tDCS DEVICE**	**TYPE OF STIMULATION**	**MEDICATION STATUS**	**STIMULATION SITE** **(ANODE/CATHODE)**	**ELECTRODE SIZE**	**INTENSITY** **(mA)**	**DURATION IN MINUTES**	**NUMBER OF SESSIONS**
PEREIRA, JB et al (2013) Phoresor, Iomed Inc.(EUA)	Crossover experiment of tDCS combined with fMRI	ON	**ANODE** F3 (DLPFC) LEFT OR P3-T5 (TPC) LEFT **CATHODE** FP2 RIGHT	**A PAIR OF ELECTRODES** **SPONGE** 35 cm2	2mA	20 MINUTES	1 SESSION (2h break between experiments)
PIETROBON et al. (2021) DC-STIMULATOR, neuroConn (Germany)	Combination of audiovisual verbal production therapy and anodal tDCS	ON	**ANODE** F7 LEFT HEMISPHERE (DRILL) **CATHODE** FP2 RIGHT	**TWO SPONGE ELECTRODES** 35 cm2	2mA	20 MINUTES	9 SESSIONS
LEYDON,C et al. (2022) Neuroelectrics Starstim (Switzerland)	Cross-over study of the immediate effects of tDCS on the premotor cortex of speech production	ON	**ANODE** F3(DLPFC) LEFT **SHAM** F3(DLPFC) LEFT	**Ag/AgCl Electrodes**	2mA	20 MINUTES	2 SESSIONS (crossover - active or sham with a 1-2 week interval)
ROSA, RR, et al. (2023) NKL Microestim Foco tDCS (Brazil)	Effects of tDCS on voice and speech in Parkinson's disease	ON	**ANODE** C3 (M1 - PRIMARY MOTOR CORTEX) **CATHODE** FP2 RIGHT.	**TWO SPONGE ELECTRODES** 7×5cm2	2mA	20 MINUTES	10 SESSIONS
JOHARI K (2024) Soterix HD-tES de 9 MxN. (New York)	Speech and voice outcomes after HD-tDCS over the left SMA	ON	**ANODE** F3(DLPFC) LEFT **CATHODE** FP2 RIGHT.	**ELECTRODES** **HD** (1.2 cm diameter) **with HD-tDCS cap**	2mA	20 MINUTES	5 SESSIONS CONSECUTIVE
BRABENEC, L et l (2024) DC-STIMULATOR PLUS (Germany)	Short-term effects of tDCS on motor speech in Parkinson's disease	ON	**ANODIC** ANODE - STG RIGHT POSTERIOR CATHODE - STG LEFT POSTERIOR **CATHODIC** CATHODE - STG RIGHT POSTERIOR ANODE - STG LEFT POSTERIOR	**TWO SPONGE ELECTRODES** 5×5cm2	2mA	20 MINUTES	3 SESSIONS ANODAL CATHODAL SHAM SEPARATED BY ONE DAY WITHOUT STIMULATION

**Caption:** tDCS - Transcranial direct current stimulation fMRI - Functional Magnetic Resonance Imaging TPC - Left Temporoparietal Cortex FP2 - Orbital Frontal Cortex DLPFC - Dorsolateral Prefrontal Cortex PFC - Prefrontal Cortex TC - Temporal Cortex M1 - Primary Motor Cortex IPFC - Inferior Prefrontal Cortex STG - Superior Temporal Gyrus mA - microampere.

### Analysis of results

For this meta-analysis, the results were compared regarding the common outcome of speech and voice studies with anodal or cathodal tDCS compared to sham or between the two groups. The mean between the groups that received stimulation and those that did not was used as a reference. We used Review Manager 5.4.1 software to calculate the treatment effect and 95% confidence intervals for the studies, using the mean, standard deviation, and sample size, analyzing the results and forest plots. We also assessed the risk of bias for the studies included in this meta-analysis: selection bias (randomization and allocation), performance bias (blinding of participants and personnel), detection bias (blinding of outcome assessments), attrition bias (incomplete results), reporting bias (selective reporting), and others. These biases were classified according to the risk of bias as low risk, unclear risk, or high risk.

**Figure 2:**
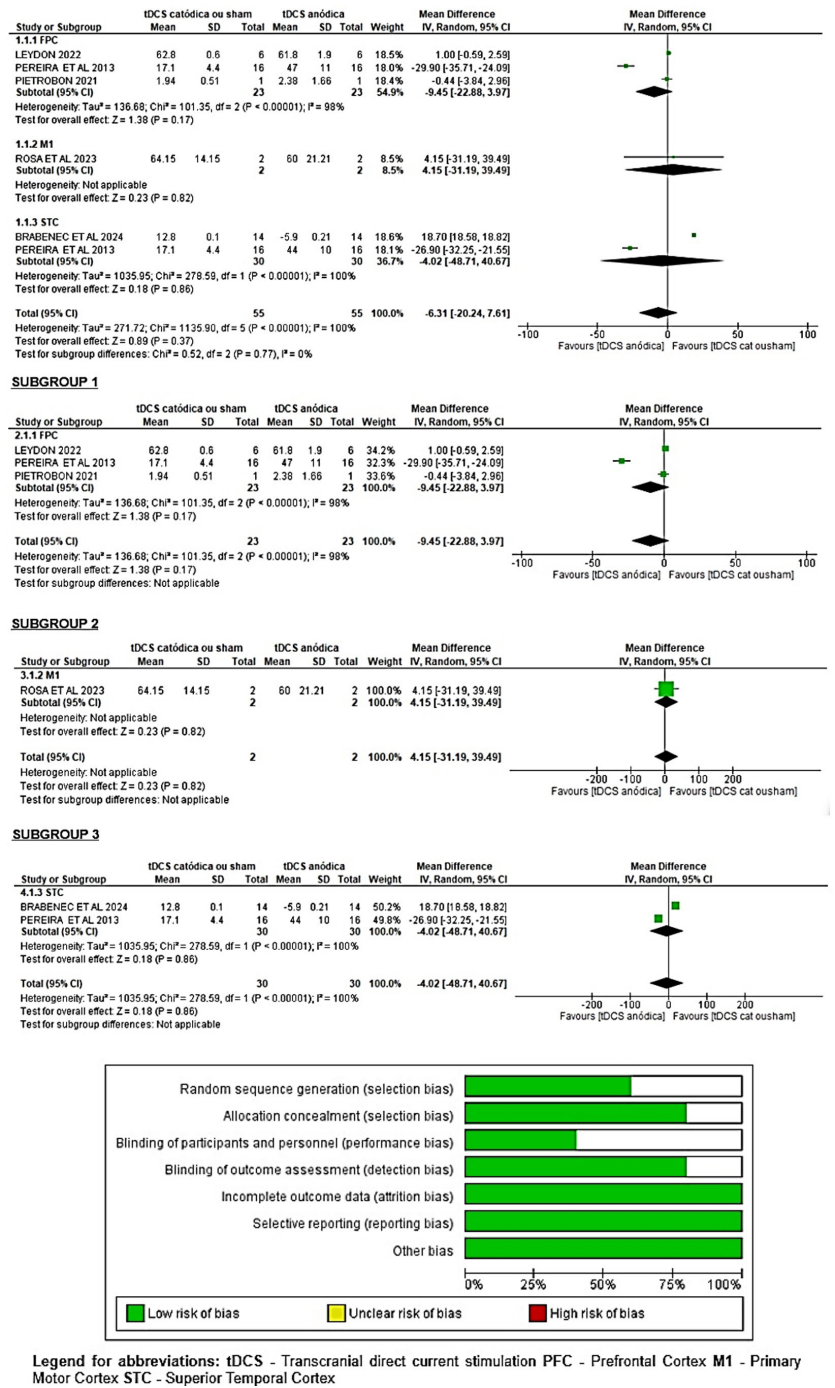
Meta-analysis of tDCS studies for the treatment of voice and/or speech in PD with target areas for Prefrontal Cortex (FPC), Motor Cortex (M1) and Superior Temporal Cortex (STC).

The meta-analysis performed with the included articles will be presented according to the target area for tDCS stimulation. We can observe in Figure 2 the effect of the treatment and the confidence intervals (95%) of the studies, through the table and forest plot of the studies with an overall effect size of Z=0.89 (P=0.37), and significance of (P<0.00001) for heterogeneity, presented by the random effects model.

**Caption:** tDCS - Transcranial direct current stimulation PFC - Prefrontal Cortex M1 - Primary Motor Cortex STC - Superior Temporal Cortex.

The forest plot showed three studies for the target area of ​​the prefrontal cortex (FPC): Leydon et al.^(25)^, Pereira et al.^(24)^ e Pietrobon et al.^(21)^; with an overall effect size of 1.38 (P = 0.17), with significance (P < 0.00001) for heterogeneity; in the target of the primary motor cortex (M1), one study (Rosa, et al; 2023) with an effect size of 0.23 (P = 0.82); and in the area of ​​the superior temporal cortex (STC), two studies: Brabenec, et al (2023) and Pereira, et al (2013), with an effect size of 0.89 (P = 0.86) and significance of (P < 0.00001) for heterogeneity. In these results, we observed that the larger the effect size, or strength of association, the greater the impact the factor under study has on the outcome. Studies targeting the FPC target area had the largest effect size and, therefore, the greatest impact on speech and voice outcomes with tDCS for PD.

High heterogeneity was observed in the meta-analysis, possibly due to differences in intervention protocols regarding the location of tDCS application and treatment duration. Subgroup analysis shows that tDCS stimulation targeting the prefrontal cortex (FPC) had the largest effect size because it corresponds to the functional cortical area responsible for motor control of voice production, via the laryngeal motor cortical pathway, with the dorsolateral prefrontal cortex (DLFPC) responsible for the learned tasks of speaking and singing, regulating the control of voice production and frequency; the ventromedial prefrontal cortex (VMFPC) with the laryngeal swallowing center for airway protection^(9)^.

In assessing the risk of bias using the RoB 2.0 tool, we observed a low risk of bias in the qualitative assessment for most studies in the domains of randomization, allocation, results, and reporting (Leydon et al., 2022; Pereira et al., 2013; and Brabenec et al., 2023). In the domain of blinding of participants and personnel, the qualitative assessment showed unclear risk for the following studies: Pereira et al. (2013), Pietrobon et al. (2021), and Rosa et al. (2023). The randomization and allocation domain also presented unclear risk for the study by Rosa et al. (2023), as it was not applicable.

**Figure 3:**
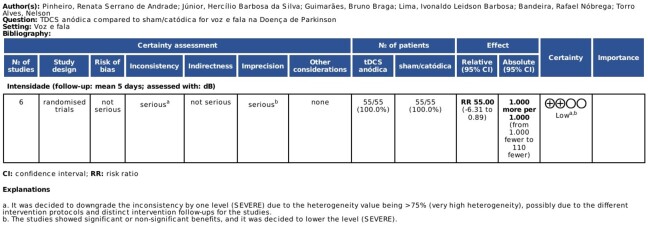
GRADEpro of tDCS studies for the treatment of voice and/or speech in PD to assess the certainty of evidence and the strength of clinical recommendations.

Finally, the online version of GRADEpro, a systematic tool for assessing the certainty of evidence and the strength of clinical recommendations, was used. After analyzing the results according to the topics described in GRADE, the studies included in this meta-analysis were classified as having ***low*** certainty of evidence, as shown in Figure 3, which describes the GRADE assessment items for this study.

Regarding *risk of bias* and *indirect evidence*, they were classified as not serious; for *inconsistency*, classified as *serious*, the inconsistency level was lowered due to the heterogeneity (>75%), possibly due to different intervention protocols and different follow-ups in the studies. For *imprecision* (serious), a lower level of evidence was warranted because the studies showed a benefit, but it was not significant. It can be concluded that future research is very likely to have a significant impact on the confidence of the effect estimate and may alter it.

Using this methodological resource ensures that evidence-based decisions are as reliable as possible, promoting better health outcomes.

## DISCUSSION

This section presents concepts of non-invasive neuromodulation through tDCS and how this therapeutic resource has impacted the rehabilitation of neurological disorders and alterations, more specifically Parkinson's disease. The theoretical and conceptual articles and publications in this SR provide us with relevant and current information on the topic.

### Systematic review

Initially, a study by Fox et al. (2014) surveyed invasive and non-invasive brain stimulation in psychiatric and neurological diseases, showing the most effective target sites and areas for each disease, according to resting-state functional connectivity. Among these, we can observe some of the stimulated areas: Alzheimer's disease (TMS or tDCS - bilateral DLPFC + parietal/temporal), Depression (TMS or tDCS - DLPFCe/DLPFCd), Pain (TMS or tDCS - M1), and Parkinson's disease - TMS or tDCS (M1, SMA), among others^(10)^.

Two studies conducted by Miterko et al.^(11)^, Manto et al.^(12)^, and collaborators, reported in a consensus and conceptual paper the use of Cerebellar Neurostimulation for PD, stating that the anatomical communication between the cerebellum and the basal ganglia provides expanded application and results of tDCS for dystonias and other neurological diseases.

Anodal cerebellar tDCS improves the results of handwriting and circle drawing tasks, and rehabilitates speech and language deficits. They also showed positive results for verbal fluency and cognitive symptoms in PD, in addition to increasing the ability to learn from mistakes and form and retain motor memory. Manto (2022) observed that, through functional neuroimaging, verbal fluency is influenced by cerebellar neurostimulation, particularly phonemic neurostimulation, through anodal cerebellar tDCS in the right posterolateral region. It was also found that there was a perception of a change in the tone of the voice of sustained vowels compared to sham, when 19 patients received cerebellar tDCS with neuronavigation, promoting smaller vocal compensations for pitch disturbance, confirming that cerebellar tDCS can modulate altered auditory-vocal integration^(11,12)^.

The application of continuous theta-burst stimulation (cTBS) and cerebellar tDCS have been demonstrated by other authors to be effective and beneficial for speech and voice. According to authors^(13)^, anodal activation of the right cerebellum by tDCS was observed in tasks of verbal generation, verbal working memory, voice production, and compensatory voice adjustment. In addition to these observed aspects, and even more important, is the causal relationship found in studies between the right cerebellum and speech production. The authors' results show that tDCS on the right cerebellum resulted in increased vocal compensation for pitch or F1 disturbances.

Studies have also shown that the use of anodal cerebellar tDCS before or during speech and voice production tasks leads to improved vocal compensation for pitch disturbances, with the magnitude and latency of vocal responses not modulated by the stimulation time. Furthermore, the modulatory effects of anodal cerebellar tDCS on vocal compensations were observed regardless of the size and direction of pitch perturbations^(14)^.

Other authors, Montemurro et al.^(15)^, Saluja et al.^(16)^, and Weismer^(17)^, conducted literature reviews on tDCS and reported current evidence on the application of neuromodulation in PD patients regarding voice and speech. They observed that tDCS favors outcomes in PD motor symptoms more prominently when associated with rehabilitation, likely due to neuronal plasticity and long-term synapse generation (LTS).

Regarding speech and language rehabilitation in PD, they consider that approximately 89% of PD patients may develop speech and voice disorders (reduced volume, monotonous voice, and breathy quality), articulation (imprecise consonants and vowel centralization), and frequency (increased, decreased, and variable). Speech-language therapy with tDCS improved sound pressure levels in sustained vowels, reduced the Voice Handicap Index score, and improved text and monologue reading. It was also observed that tDCS applied to jaw movements associated with speech, syllable repetition, and chewing reinforced the concept that the effects of tDCS depend on the task, as it showed a negative relationship between nonverbal oromotor performance and speech production measures^(15-17)^.

In addition to the cerebellar target area for vocal pitch regulation, many tDCS neuromodulation studies focus on auditory feedback for speech motor control, as this aspect provides sensory information that allows monitoring and adjusting vocal output to produce intended speech goals. This control process is known as auditory-motor interaction, manifested by compensatory adjustments in response to mismatches between the expected auditory feedback and that produced in the fundamental frequency (F0), intensity, or first formant (F1) of the voice^(18)^.

These authors^(18)^ used anodal tDCS in 17 healthy adults to modulate the cortical excitability of the left DLPFC and examine its effects on auditory-motor integration for vocal pitch regulation. Participants vocalized vowel sounds while listening to their voices pseudo-randomly shifted in frequency to higher or lower Hz, during or after active or sham anodal tDCS over the left DLPFC. In the results, active anodal tDCS over the left DLPFC led to significantly lower peak magnitudes and shorter peak times of vocal compensations for pitch perturbations than sham stimulation.

Finally, the two systematic reviews by Baig et al.^(19)^ and Balzana et al.^(20)^ used database searches to verify the evidence on the usefulness of non-invasive neuromodulation by tDCS in the treatment of dysarthria. The databases searched and keywords for Baig's (2022) review were: Google Scholar, Embase, Scopus, PubMed, Cochrane, and Web of Science, keywords (dysarthria, tDCS, TMS, neurological disorder), with 9 studies with TMS and 5 with tDCS. Speech-language therapy associated with tDCS was offered in 6 studies, and positive effects of tDCS were found in 83% of the studies. The significant post-treatment difference between the sham and treatment groups was associated with the sequential movement rate (Hedges' g = 0.79, 95% CI [0.075, 1.50] P = 0.03) favoring tDCS^(19)^.

And the RS by Balzana et al.^(20)^ had the following searches: Medline, PsycInfo, Embase, Linguistics and Language Behavioral Abstracts, Web of Science and Cochrane, keywords (Brain and electrical stimulation, Dysarthria and Research design), finding 10 studies (6 randomized controlled trials and 4 crossover studies) focused on Neuromodulation (6 TMS, 3 tDCS and 1 tACS) in 268 adults with PD, stroke and neurodegenerative cerebellar ataxia, related to dysarthria. The 3 studies provided anodal stimulation over the cerebellum or M1, with a current intensity of 2 mA. tDCS was administered for 20 (n = 2) or 30 (n = 1) minutes. In all trials, stimulation was administered 5 times a week for a total of 10 sessions, and showed that the evidence for the use of tDCS in the treatment of dysarthria needs more randomized clinical trials so that its results can be replicated more effectively^(20)^.

### Meta-analysis

This review also selected six empirical articles for inclusion in the meta-analysis, four clinical trials, and two case reports regarding speech and voice symptoms presented before and after the application of tDCS for PD in the target areas of the prefrontal cortex (FPC), motor cortex (M1), and superior temporal cortex (STC).

Pietrobon et al.^(21)^ and Rosa et al.^(22)^ conducted clinical case studies with the following methods and results. Pietrobon et al. (2021) stimulated Broca's area in the left inferior prefrontal cortex (F7) with anodal tDCS, 2 mA for 20 minutes, in 9 sessions, using the 10-20 system in a 79-year-old patient with PD and chronic nonfluent post-stroke aphasia and severe naming deficit, combined with audiovisual therapy for naming common objects using videos. The results showed a significant increase in naming scores between pre- and post-treatment^(21)^.

Rosa et al. (2023) verified the effects of tDCS on voice and speech in PD by studying two male patients with PD and hypokinetic dysarthria. They applied 10 sessions of anodal tDCS to the primary motor cortex (M1) using the 10-20 system (C3) and cathodal tDCS to the primary motor cortex (FP2), with an intensity of 2 mA, for 20 minutes, on 10 consecutive days. One patient received speech-language therapy concomitantly with neuromodulation, and the other did not. The authors observed improvements in phonation time, velar movement, and acoustic analysis parameters (frequency, jitter, shimmer, and noise) for the participants and concluded that conventional speech-language therapy combined with tDCS produces better speech and voice outcomes than tDCS alone^(22)^.

Johari et al. (2024) have not yet presented results in their study. After a complete reading, it was observed that it was a clinical trial protocol for the application of HD-tDCS on the left supplementary motor area (SMA), where the objective is to investigate the immediate and short-term effects of HD-tDCS on speech and voice deficits. The intention is to pair 24 participants with PD and 24 controls with the use of anodal HD-tDCS at 2 mA, for 20 minutes, on 5 consecutive days in the left SMA. This is a randomized clinical trial protocol registered with Cochrane and scheduled to start in 2024^(23)^.

And the three with randomized clinical trials were those of the authors: Pereira et al.^(24)^, Leydon et al.^(25)^, and Brabenec et al.^(26)^ with the application of tDCS in brain areas for speech and voice in PD. Pereira et al. (2013) verified the modulation of verbal fluency networks by tDCS in Parkinson's disease. They evaluated the effects of tDCS on functional networks of phonemic and semantic fluency in 16 patients with idiopathic PD, applying assessment, cognitive, and depression scales, followed by the application of anodal tDCS over the left DLPFC or TPC, and cathodal over the right FP2, with an intensity of 2 mA, for 20 minutes, in a session with a 2-hour interval between experiments. They found that tDCS improved functional connectivity in verbal fluency when applied to the DLPFC more than to the TPC in PD^(24)^.

Another author, Leydon et al.^(25)^, observed speech changes in individuals diagnosed with PD and evaluated the impact of tDCS. The study consisted of six men with PD, aged between 65 and 78 years, with a double-blind, crossover, and controlled study design, investigating the immediate effects of tDCS on the premotor cortex on speech production. The left DLPFC was stimulated with 2 mA for 20 minutes with anodal and sham tDCS, with a one- or two-week interval between stimulation. The following parameters were evaluated before and after active and sham stimulation: auditory perceptual evaluation (AVE) and acoustic voice results (fundamental frequency, intensity, NHR, and speech rate). The results found speech rate below normal for participants before tDCS, and after neuromodulation, there was a significant increase in speech rate in conversation after stimulation, which did not occur with sham tDCS. The NHR increased after stimulation, but the other measures of perceptual disturbance, fundamental frequency or intensity did not show a statistically significant difference.

Finally, the study by Brabenec et al. (2024) analyzed the short-term effects of HD-tDCS on motor speech in PD. This pilot study involved remote treatment combined with LSVT^®^. The study population consisted of 14 patients with diagnosed PD, medication-on status, and dysarthria symptoms. Three sessions of tDCS (anodal, cathodal, and sham) were administered at an intensity of 2 mA for 20 minutes, separated by one day, and speech tasks with 150 words were administered. The results showed a significant effect of stimulation in improving the median duration of inappropriate silences in speech, which, when altered, negatively impacts speech rhythm and fluency. The authors concluded that anodal tDCS targeting auditory feedback can improve motor speech fluency in individuals with PD^(26)^.

The results were described by conceptual and literature review/systematic studies in this SR, highlighting the benefits and effectiveness of tDCS. Regarding empirical studies, most of them involved the application of anodal tDCS to specific areas of the brain, with an intensity of 2 mA for 20 minutes. The greatest effect was observed in the prefrontal cortex, as verified in the meta-analysis, for the effects and evidence of voice and speech, with improvements in sustained vowel sound pressure levels, verbal fluency (phonemic), word reading, and monologue.

### Limitations and Perspectives of the Study

Among the limitations found in this meta-analysis, there are still some knowledge gaps that need to be clarified regarding the protocols used: the amount and duration of stimulation, and the most appropriate target areas to enhance voice and speech outcomes with tDCS in PD.

Currently, there are few studies in this area, and we can observe the presence of possible biases in the studies, due to the heterogeneity found in the meta-analysis. This highlights the need for more robust and well-controlled studies to confirm the benefits and results already found in the scientific literature, as well as in clinical practice for the study population.

It is necessary to promote larger studies and randomized clinical trials with better methodological designs that can more accurately investigate the results and duration of the effects of tDCS associated with voice and speech therapeutic resources for PD.

## CONCLUSION

After observing the findings of this SR with a meta-analysis on the use of tDCS for speech and voice disorders in individuals with Parkinson's, it is clear that tDCS is a therapeutic resource that has been implemented in the treatment of PD for some years, as a complement to therapies and as an alternative to other invasive or non-invasive therapies for Parkinson's, such as Deep Brain Stimulation (DBS) surgery or Transcranial Magnetic Stimulation (TMS), among others.

The meta-analysis studies targeting the prefrontal cortex, primary motor area, and superior temporal region showed the best evidence and impact on speech and voice outcomes with the use of tDCS for PD, primarily by providing auditory feedback regulation in the motor activity of speech and voice, regulating parameters such as sound pressure, fundamental frequency (F0), and first formant (F1), as well as verbal fluency and word reading. It is concluded that the target areas stimulated with tDCS can bring benefits through functional connectivity and connections established for the production and regulation of speech and voice in PD.

The SR and meta-analysis on this topic are encouraging and establish models that can facilitate the development of clinical trials aimed at supporting the application of tDCS in the treatment of speech and voice in PD, and which, based on scientific evidence, consolidates it as a viable therapeutic option in speech therapy.
