# Supplementary material for: Effects of Transcranial Direct Current Stimulation on Speech and Voice Changes in Parkinson's Disease: a Systematic Review with Meta-Analysis
Source: Codas. 2026 Feb 27;38(1):e20250002. doi: 10.1590/2317-1782/e20250002en (PMC12978332; doi:10.1590/2317-1782/e20250002en)
Supplement: Translation

# Translation: Efeitos da Estimulação Transcraniana por Corrente Contínua nas alterações de Fala e Voz para a Doença de Parkinson: uma Revisão Sistemática com Metanálise

**DOI:** 10.1590/2317-1782/e20250002pt

**Authors:** Renata Serrano de Andrade Pinheiro, Hercílio Barbosa da Silva, Bruno Braga Guimarães, Ivonaldo Leidson Barbosa Lima, Rafael Nóbrega Bandeira, Nelson Torro Alves

**Affiliations:** 1 Programa de Pós-graduação em Neurociências Cognitiva e Comportamento – PpgNeC, Departamento de Psicologia, Universidade Federal da Paraíba – UFPB - João Pessoa (PB), Brasil.; 2 Departamento de Pesquisa do Neuronus, Instituto Transdisciplinar de Ensino e Pesquisa sobre o Cérebro - Goiânia (GO), Brasil.; 3 Departamento de Fonoaudiologia, Universidade Federal do Rio Grande do Norte – UFRN, Natal (RN), Brasil.; 4 Departamento de Fonoaudiologia, Universidade Federal da Paraíba – UFPB - João Pessoa (PB), Brasil.

**Keywords:** Doença de Parkinson, Estimulação Transcraniana por Corrente Contínua, Voz, Qualidade Vocal, Fala

## Abstract

**Objetivo:**

Esta metanálise tem o objetivo de avaliar a evidência existente dos efeitos da tDCS nas alterações de fala e de voz de pacientes com DP.

**Estratégias de Pesquisa:**

PubMed, LILACS, EMBASE, Cochrane Central Register of Controlled Trials, Science Direct, Web of Science, Scopus e de literatura cinzenta Google Scholar e Open Grey. A busca incluiu os descritores: “Parkinson Disease, Transcranial Direct Current Stimulation, Voice, Speech” combinados com AND e OR.

**Critérios de Seleção:**

Pacientes com a Doença de Parkinson, ambos os sexos. Uso da Estimulação Transcraniana por Corrente Contínua (tDCS) para tratar os parâmetros de voz e fala nos pacientes acometidos pela Doença de Parkinson.

**Análise de dados:**

Um total de 1.345 artigos, 14 artigos incluídos na revisão sistemática e 6 artigos na metanálise. O risco de viés e o nível de evidência foram avaliados pelo software REVIEW MANAGER 5.4.1. e GRADE.

**Resultados**

Os resultados mostraram um tamanho de efeito geral da tDCS de Z= 0.89 (P=0.37). Estudos com área alvo no córtex pré-frontal (PFC) apresentaram maior tamanho de efeito 1.38(P=0.17), portanto com maior impacto para desfecho de fala e voz com uso de tDCS para DP. Três estudos apresentaram *baixo risco de viés* e três estudos apresentaram risco pouco claro.

**Conclusão:**

Apesar do baixo número de estudos, os achados desta metanálise sugerem a potencial aplicabilidade da tDCS como ferramenta adjuvante no tratamento das alterações de voz e fala em pacientes com DP.

## INTRODUÇÃO 

O aumento da expectativa de vida e o avanço no diagnóstico e tratamento das doenças degenerativas que se apresentam, principalmente, acima dos 65 anos, têm indicado a Doença de Parkinson(DP) como uma das doenças de maior incidência na atualidade^(1)^. A DP é caracterizada como uma degeneração de neurônios na substância negra do mesencéfalo, que provoca uma diminuição na produção do neurotransmissor dopamina. A fisiopatologia dos sinais clássicos da DP inclui a disfunção nos circuitos dos gânglios da base como um fator determinante para o surgimento de alterações no controle do movimento, pelos sintomas motores e não motores, com a presença de disfunções da voz e da fala, caracterizada pela Disartrofonia e/ou Disartria^(2)^.

Os mecanismos de feedback motor e sensorial de feedforward compõem os atuais modelos de fala e propõem a função complexa do cérebro ao desempenhar este mecanismo. Os efeitos prejudiciais nos mecanismos de controle motor da voz na DP ocorrem devido a degeneração dos neurônios dopaminérgicos, apresentando prejuízos nos mecanismos do movimento que desenvolvem os distúrbios motores na fala: tremor na voz, volume de voz reduzido e dificuldades de entonação da voz^(3)^. As alterações no controle motor da voz e fala na DP são evidenciados pela diminuição da entrada excitatória para o córtex, o que provoca e dá origem aos déficits fisiológicos nos sistemas periféricos (laríngeo, articulatório e respiratório), como também diminui a atividade cortical na rede de produção da fala^(4)^.

Nos últimos anos é crescente o número de pesquisadores que têm se dedicado a estudar os efeitos de técnicas de estimulação cerebral não-invasivas, como a Estimulação Transcraniana por Corrente Contínua (tDCS) no manejo das dificuldades causadas pela DP. A tDCS é uma técnica em que uma corrente elétrica de baixa intensidade é ministrada através de dois eletrodos de superfície – um ânodo e um cátodo – posicionados no escalpo^(5)^. Esta corrente pode induzir mudanças no potencial de repouso da membrana neuronal, modulando a sua excitabilidade e tornando a região cerebral abaixo dos eletrodos mais ou menos ativa, a depender da polaridade da corrente. A aplicação dessa estimulação por repetidas vezes a uma determinada região alvo no cérebro pode modular as redes neurais subjacentes através do mecanismo de neuroplasticidade pós-sináptica, produzindo mudanças funcionais ou comportamentais significativas^(5)^.

Apesar do crescente número de estudos com tDCS no tratamento de funções motoras e não-motoras em indivíduos com DP, ainda há lacunas e questões a serem respondidas sobre a eficácia desse recurso terapêutico nesta população. Até onde se sabe, ainda não há nenhum estudo sobre os efeitos da tDCS nas alterações de voz e fala causados pela DP. Por este motivo observamos a importância de se realizar uma Revisão Sistemática nas bases científicas e verificar por meio da Metanálise de qual maneira o recurso da Neuromodulação por tDCS está trazendo benefícios e potencializando os resultados terapêuticos da Fonoaudiologia na voz e fala de indivíduos com DP.

O objetivo deste estudo foi realizar uma revisão sistemática com metanálise dos artigos que fazem uso da Estimulação Transcraniana por Corrente Contínua como recurso tecnológico adjuvante para o tratamento de fala e voz em indivíduos com Doença de Parkinson.

## MÉTODO

Esta Revisão Sistemática teve o seu protocolo cadastrado na Plataforma International Prospective Register of Systematic Reviews (PRÓSPERO) antes da sua realização, com o número CRD42024542291, após pesquisa e verificação de ausência de estudos de RS com Metanálise que contemplam essa temática e avaliação

### Desenho do estudo

Neste protocolo foram incluídos os seguintes aspectos relacionados ao tema da pesquisa: a pergunta da pesquisa, os critérios para seleção e busca dos artigos nas bases de dados exploradas, a elegibilidade, a extração dos dados, a análise dos dados e a forma que os desfechos foram avaliados. Não houve limitação quanto ao idioma ou ano de publicação nos artigos incluídos nesta RS. O acrônimo PICOS (Patient, Intervention, Comparator, Outcomes e Study design) foi utilizado para a pergunta norteadora de pesquisa desta revisão sistemática segundo o protocolo PRISMA: “Quais os efeitos e evidências na voz e fala de indivíduos acometidos pela Doença de Parkinson que utilizaram a estimulação transcraniana por corrente contínua-tDCS como recurso terapêutico?”.

Os artigos incluídos nesta RS foram limitados quanto ao idioma (português, inglês ou espanhol) e quanto ao ano de publicação foram dos últimos dez anos. Foram selecionados de acordo com os critérios de inclusão e exclusão dos estudos. A coleta de dados da RS nas bases de dados foi realizada no período de 01.05.2024 à 31.07.2024.

A descrição dos critérios utilizados pelo acrônimo PICOS para esta RS está disposta na Tabela 1 abaixo. A tabela também aborda os critérios de inclusão e exclusão dos estudos nesta RS.

**Tabela 1:** Critérios PICOS para a seleção de estudos desta Revisão Sistemática.

	INCLUSÃO	EXCLUSÃO
PATIENT	Pacientes com a Doença de Parkinson, ambos os sexos, nos estágios 2 ou 3 da escala de Hoehn e Yahr da DP.	-Condição de saúde individual: hemodinâmica instável, outra doença neurológica, ou doença psiquiátrica não tratada.
(População)		
		- Uso de DBS ou metal no cérebro, ou algum motivo que impeça a tDCS.
		-Alteração auditiva ou uso de AASI ou implante coclear.
INTERVENTION	Uso da Estimulação Transcraniana por Corrente Contínua (tDCS) para tratar os parâmetros de voz e fala nos pacientes acometidos pela Doença de Parkinson.	Os estudos que não utilizem tDCS no tratamento proposto.
(Intervenção)		
	(a-tDCS, c-tDCS, s-tDCS ou sham)	
COMPARISON	Áreas de aplicação do Protocolo no Sistema 10-20 para a tDCS em foco na voz e fala para indivíduos com Doença de Parkinson.	Estudos que não identifiquem a área estimulada com a tDCS.
(Comparação)		
OUTCOME	Melhora na produção de voz, e parâmetros acústicos, fluência e inteligibilidade de fala, diminuição de sinais e sintomas de alteração na voz e fala.	Estudos que não tenham desfechos para voz e fala.
(Desfechos)		
STUDY DESIGN	Estudos de intervenção (transversal ou longitudinal), com ensaios clínicos randomizados ou não randomizados, outros estudos que abordem a Estimulação Transcraniana por Corrente Contínua (tDCS) em indivíduos com DP.	Acurácia, Prevalência, Prognóstico.
(Desenho do Estudo)		
DATA REPORT	Utilizar dados que permitam analisar e estimar os efeitos da tDCS associados ao treinamento ou avaliação de voz e fala em DP.	
(Relatório de Dados)		
TYPE OF PUBLICATIONS	Deve ter sido publicado em periódico revisado por pares nos últimos dez anos e em qualquer idioma.	
(Tipo de Publicações)		

**Legenda: tDCS -** Transcranial direct current stimulation **(**Estimulação Transcraniana por Corrente Contínua) **atDCS -** Estimulação Transcraniana por Corrente Contínua Anódica **ctDCS -** Estimulação Transcraniana por Corrente Contínua Catódica **sham ou stDCS -** Estimulação Transcraniana por Corrente Contínua simulada **DP -** Doença de Parkinson **DBS -** Deep Brain Stimulation (Estimulação Cerebral Profunda) **AASI -** Aparelho de Amplificação Sonora Individual.

### Pesquisa na literatura e critérios de seleção 

Inicialmente foi realizada a busca e identificação dos termos e descritores a serem utilizados na plataforma de Descritores em Ciências da Saúde - DeCS e Medical Subject Heading - MeSH para o tema da pesquisa (Parkinson), o recurso terapêutico (Estimulação Transcraniana por Corrente Contínua - tDCS) e os desfechos previstos dos estudos incluídos nesta RS para fala e voz em indivíduos com Parkinson sob essa atividade terapêutica.

No planejamento da estratégia de pesquisa foram encontrados e utilizados os termos e descritores para realizar a revisão sistemática. Os descritores utilizados nesta revisão foram:“Parkinson Disease”, “Transcranial Direct Current Stimulation” “Voice”, e “Speech” e seus correspondentes em língua portuguesa “Doença de Parkinson”, “Estimulação Transcraniana por Corrente Contínua”, “Voz”, e “Fala”. Foram utilizados com os boleadores AND e OR de acordo com as combinações entre os descritores nas bases de dados pesquisadas.

As buscas e pesquisa dos termos e descritores foram realizadas nas seguintes bases de dados: PubMed, Cochrane Central Register of Controlled Trials, Embase, Scopus, Web of Science, Scielo, Lilacs, Science Direct, Medline. Participaram da pesquisa também as bases de dados de literatura cinzenta Google Schoolar e Open Grey.

### Coleta e análise de dados

Foi utilizado para essa Revisão Sistemática o Preferred Reporting Items for Systematic Reviews and Meta-Analyses (PRISMA-2020)^(6)^, este protocolo é o recomendado por considerar uma melhor assertividade na descrição e relato das revisões sistemáticas.

Com o término da busca nas bases de dados, os artigos da RS foram exportados para o gerenciador de referências ENdNote^®^ (EndNote / Clarivate Analytics, PA, EUA) com a sua organização e remoção dos artigos duplicados. Em seguida, os artigos foram exportados para o software Rayyan^®^ (Qatar Computing Research Institute, Doha, Qatar) quando aconteceu a seleção dos estudos de forma cega e independente por dois revisores, com manutenção dos registros das decisões na plataforma e resolução de discordâncias por consenso. Os artigos foram selecionados de acordo com os critérios de elegibilidade e quanto aos critérios de inclusão e exclusão dos estudos.

Após a finalização das etapas de coleta dos dados, análise, interpretação e discussão dos resultados, foi seguido o PRISMA^(6)^ a fim de elaborar o relatório final dessa Revisão Sistemática sobre os Efeitos e Evidências da Estimulação Transcraniana por Corrente Contínua nas alterações de Voz e Fala em indivíduos com DP. A Metanálise seguiu o modelo de efeitos aleatórios para os modelos estatísticos aplicados através do software REVIEW MANAGER 5.4.1 . Foi realizada a análise dos subgrupos para avaliar a robustez dos resultados, observada nos gráficos de forest plot.

### Avaliação da qualidade

A qualidade metodológica dos artigos da pesquisa foi realizada seguindo as diretrizes que auxiliam a elaboração de uma RS de boa qualidade: *The Cochrane Reviewers Handbook* ^(7)^. Será utilizado para a ***avaliação do risco de viés*** , a ferramenta RoB 2.0^(8)^ (Revised Cochrane risk-of-bias tool for randomized trials) recomendada pela Cochrane para avaliar o risco de viés de ensaios clínicos randomizados, observando os domínios: viés no processo de randomização, desvios da intervenção pretendida, devido a dados faltantes, na aferição dos desfechos, e no relato dos desfechos^(7)^ e para a ***avaliação do*** ***tamanho do*** ***efeito*** , o software REVIEW MANAGER 5.4.1, que mostra o forest plot com tamanho do efeito dos estudos, grau de confiança e heterogeneidade que o resultado do estudo pode apresentar. A precisão dos tamanhos de efeito foram estimados utilizando um intervalo de confiança de 95%.

## RESULTADOS

### Extração dos dados

O diagrama PRISMA^(5)^, descreve as etapas da Revisão Sistemática, nele podemos observar os resultados através dos seguintes tópicos: **Identificação** onde foram encontrados na diferentes bases de dados 1.345 artigos nos seguintes locais: PubMed, Cochrane Central Register of Controlled Trials, Embase, Scopus, Web of Science, Scielo, Lilacs, Science Direct, Medline e nas bases de dados de literatura cinzenta Google Scholar e Open Grey. No tópico **Triagem** foi realizada a exportação dos 1.345 artigos para o Rayann para a realização da seleção e screening destes artigos pela leitura dos títulos e resumos. Foram removidos 11 artigos duplicados do total de artigos da busca pelo EndNote após seleção automática no Rayyan. Os artigos excluídos por não fazer parte do tema da pergunta de pesquisa foram 1.279 artigos, ficando um total de 66 artigos para a leitura completa seguindo os critérios de elegibilidade da busca nas bases de dados proposta no Protocolo de Revisão Sistemática do PRÓSPERO. No final, foram analisados 14 artigos para seleção e análise de resultados descritos em forma de Tabelas 2 e 3, com a realização da Metanálise pelo tópico **Incluídos** do PRISMA(2020) para esta RS, realizando a exclusão dos artigos com fonte(n=1), resultados(n=2) ou comparador(n=5) que não preenchem os requisitos para a Metanálise, alguns estudos apresentavam outra população, não utilizavam a Estimulação Transcraniana por Corrente Contínua(tDCS) como recurso tecnológico ou apresentaram outro tipo de desfecho na intervenção de tratamento que não eram para voz ou fala. Ao final da análise e exclusões ficaram incluídos 6 artigos para a Revisão Sistemática com Metanálise.

**Tabela 2:** Descrição de artigos Teóricos ou Conceituais e de Revisão Sistemática com tDCS para o tratamento de voz e/ou fala em DP.

**AUTORES/ANO**	**REVISTA / DOI DA PUBLICAÇÃO**	**TIPO DE ESTUDO / TÉCNICA DE TRATAMENTO**	**OBJETIVO**	**MÉTODO / PARÂMETROS DE tDCS**	**PRINCIPAIS ACHADOS**	**CONCLUSÃO**
FOX, MD; BUCKNER, RL et al (2014)	PNAS PLUS **DOI:** 10.1073/pnas.1405003111	**A.CONCEITUAL OU TEÓRICO** Estimulação cerebral invasiva e não invasiva em doenças psiquiátricas e neurológicas	Identificar doenças tratadas com estimulação invasiva e não-invasiva, listar os locais de estimulação considerados ser mais eficaz em cada doença por ressonância magnética de conectividade funcional em estado de repouso.	- A pesquisa bibliográfica revelou 14 diferentes doenças psiquiátricas ou neurológicas com relatório publicado de eficácia tanto para estimulação cerebral invasiva e estimulação cerebral não invasiva. - Correlações entre regiões de DBS e todos os outros voxels cerebrais foram computados e relacionados aos locais com evidências de eficácia como alvos para estimulação cerebral não invasiva (TMS ou ETCC).	- **Doenças e áreas de estimulação:** **Doença de Alzheimer** - DBS(Fórnix) TMS ou ETCC(DLPFC bilateral- +-parietal/temporal **Depressão** - DBS (Subgenual, VC/VS, NA, MFB, habenula) TMS ou ETCC(DLPFC e , DLPFC d) **Epilepsia** - DBS(Tálamo (AN, CM), MTL) TMS ou ETCC(Foco ativo de EEG, cerebelo) **Dor** - DBS(PAG, tálamo (VPL/VPM)) TMS ou ETCC(M1) **Doença de Parkinson** - DBS( STN, GPi) TMS ou **ETCC(M1, SMA) ;** **e outras.**	Este trabalho apoia uma rede perspectiva para compreender e tratar doenças neuropsiquiátricas, destacando o potencial terapêutico do cérebro como alvo de modulação de rede.
MITERKO, LN ; BAKER, KB et al (2019)	The Cerebellum **DOI:** 10.1007/s12311-019-01041-5	Artigo de Consenso: Neuroestimulação Experimental do Cerebelo	Relatar as técnicas mais avançadas para manipulação de circuitos cerebelares em humanos e modelos animais e definir os principais obstáculos e questões para avançar.	-Foram apresentados os paradigmas recentes de estimulação animal e humana que utilizaram o cerebelo, com a identificação dos principais sucessos e fracassos,que são críticas essenciais para melhorias na terapia humana. -Foram delineados obstáculos importantes e sugestões de possíveis formas de superá-los.	- As comunicações anatômicas recentemente descritas entre o **cerebelo** e os **gânglios da base** ampliam as aplicações potenciais da ETCC no Cerebelo nas distonias e outras doenças - A **tDCS cerebelar** modifica a excitabilidade do córtex cerebelar com **efeitos colaterais menores.** - A **tDCS anódica cerebelar** melhora a cinemática de tarefas de **caligrafia** e **desenho de círculos** em pacientes com distonia de escrita, e promove a **reabilitação da fala** e dos **déficits de linguagem**.	- A tDCS anódica aumentou a capacidade de aprender com erros, além de formar e reter a memória do ato motor. O cerebelo também tem uma ampla influência e dá uma forte contribuição para domínios não motores, como a cognição. - Resultados positivos dos efeitos da tDCS anódica na fluência verbal e na percepção da dor em indivíduos saudáveis e sintomas cognitivos em pacientes com Parkinson.
MANTO,M, et al (2022)	The Cerebellum **DOI:** 10.1007/s12311-021-01344-6	Novas direções e próximos passos da abordagem não invasiva de Estimulação Cerebral do Cerebelo na saúde e na doença	Relatar os avanços feitos no uso do NIBS cerebelar e chegar a um consenso sobre os passos futuros para avançar.	- Uso da TMS para explorar a neurofisiologia cerebelar; conhecimento atual sobre tDCS cerebelo-cerebelar; o papel dos modelos animais nas aplicações cerebelares de NIBS; aplicação clínica de NIBS cerebelar (aprendizagem motora, recuperação de acidente vascular cerebral, funções de fala e linguagem, distúrbios neuropsiquiátricos e de movimento e síndromes dolorosas).	- A **neuroimagem funcional** mostrou nas pesquisas sobre **neuroestimulação** um papel cerebelar na **fluência verbal**. A **tDCS cerebelar anódica** (cerebelo póstero-lateral direito) **melhorou a fluência fonêmica**. Lin et al. - 19 pacientes com SCA foram submetidos a **cTBS** neuronavegador (cerebelo direito vs. estimulação simulada) produzindo **vogais sustentadas** enquanto percebiam a **mudança de tom de sua voz**. Em relação ao sham, o cTBS cerebelar levou a menores magnitudes de compensações vocais para perturbações de pitch, mostrando que o NIBS cerebelar pode **modular a integração auditivo-vocal anormal na SCA.**	A estimulação cerebelar não invasiva representa uma ferramenta promissora para fins terapêuticos, tanto em condições patológicas motoras, cognitivas e psiquiátricas. Direcionar a tDCS ao cerebelo para afetar indiretamente as atividades corticais e subcorticais pode ser eficaz no alívio dos sintomas de diversas patologias, bem como na melhoria das funções cognitivas ou da aprendizagem motora em indivíduos saudáveis.
MONTEMURRO,NALIAGA,N et al (2022)	International Journal of Environmental Research and Public Health **DOI:** 10.3390/ijerph19148799	Novos alvos e novas tecnologias no tratamento da doença de Parkinson	O objetivo desta revisão narrativa é mostrar novos alvos na cirurgia DBS, bem como novas tecnologias que estão em estudo e têm mostrado resultados promissores até o momento.	Revisão de literatura nas bases de dados para uma visão geral dos novos alvos de recursos terapêuticos para a DP, bem como suas limitações. Revisar pesquisas que se tornarão tratamentos eficazes e rotineiros para a DP em um futuro próximo.	- **Modulação não-invasiva da atividade neuronal espontânea** com corrente elétrica (1–2 mA) por dois ou mais eletrodos no couro cabeludo para a **regulação dos potenciais de membrana neuronal, alterações em neurotransmissores**, **glia** e **microvasos**. - Fornece **plasticidade neuronal**, pela **geração de sinapses de longo prazo** (BDNF) - A (tDCS) **afeta** os **sintomas motores da doença**, sendo os **resultados mais proeminentes** relacionados à **reabilitação**.	O progresso no desenvolvimento de dispositivos se reflete em tratamentos cada vez mais personalizados e na abordagem de novos alvos. Os novos dispositivos oferecem a vantagem de serem de menor custo e minimamente invasivos ou não-invasivos.
SALUJA, A; GOYAL,V; DHAMIJA, RK (2022)	Indian Academy of Neurology - Neuro‑Rehabilitation Supplement **DOI:** 10.4103/aian.aian_164_22	Terapia de reabilitação multimodal na doença de Parkinson e doenças relacionadas	Avaliar e resumir as evidências atuais sobre estratégias de reabilitação na DP e nas síndromes parkinsonianas atípicas. Destacar evidências e avanços na reabilitação de pacientes com parkinsonismo	-Pesquisa bibliográfica realizada na **MEDLINE (PubMed) e Cochrane**- Foram revisados os resultados relevantes do **texto completo** e os **resumos** dos estudos. Ensaios clínicos randomizados (ECR), protocolos de ensaios, diretrizes de consenso, estudos de coorte, revisões sistemáticas, metanálises, artigos de revisão, estudos pilotos, relatos de casos, estudos observacionais e estudos pré-pós.	- **Reabilitação de Fala e Linguagem em DP** - 89% dos pacientes com DP podem desenvolver **distúrbios da fala** e **voz** (redução do volume, voz monótona e qualidade soprosa), **articulação** (consoantes imprecisas e centralização vocálica) **e frequência** (aumentada, diminuída e variável) A **Terapia fonoaudiológica** melhorou os níveis de pressão sonora durante a fonação sustentada, reduziu as pontuações do **índice de desvantagem vocal** e melhorou a **leitura** da passagem do arco-íris e do monólogo.	A incorporação de estratégias de reabilitação, particularmente no início do curso da doença, pode melhorar os sintomas motores e não motores, juntamente com a Qualidade de Vida dos pacientes com DP.
WEISMER, G et al (2023)	Brain Sciences **DOI:** 10.3390/brainsci13050768	Desempenho não-verbal oromotor e controle motor da fala: teoria e revisão de evidências empíricas	Delinear questões gerais relativas às tarefas oromotoras não faladas e sua relação com a produção da fala	- Foram encontrados **12 estudos** sobre as relações entre desempenho oromotor não verbal e medidas de produção de fala em indivíduos saudáveis (3 com resultados positivos; 2 com resultados mistos 7 com resultados negativos). **- 19 estudos** sobre as relações entre desempenho oromotor não verbal e medidas de produção de fala em distúrbios de fala de base neurológica (3 com resultados positivos 5 com resultados mistos e 11 com resultados negativos).	**Estimulação transcraniana por corrente contínua (tDCS)** aplicada aos movimentos da mandíbula associados à fala, repetição máxima de sílabas e mastigação. A **relação** entre o desempenho oromotor não verbal e as medidas de produção de fala foi **negativa**. Como resultado mostrou que **os efeitos da tDCS dependem da tarefa.**	Para avançar na pesquisa e nas disciplinas clínicas do controle motor da fala, o estudo da produção da fala e seu objetivo de fornecer aos ouvintes sinais acústicos linguisticamente relevantes e variáveis no tempo é necessário e (acredito) suficiente.
BAIG, FN (2023)	Brain Stimulation DOI:10.1016/j.brs.2023.01.354	**B.REVISÃO SISTEMÁTICA** RS: Atualização sobre a eficácia terapêutica da estimulação cerebral não invasiva para a recuperação da disartria: uma revisão sistêmica	Coletar evidências da utilidade do NIBS no tratamento da disartria por meio da avaliação sistêmica de artigos originais de pesquisa.	**BASES DE DADOS:** Google Scholar, Embase, Scopus, PubMed, Cochrane e Web of Science. **PALAVRAS CHAVES:** Disartria, tDCS, EMT, distúrbio neurológico	Quatorze dos 9244 estudos foram incluídos. 9 utilizaram a TMS e 5 avaliaram a tDCS. A **terapia fonoaudiológica** adjuvante à **SBNI** foi oferecida em 6 estudos. A **disartria** resultou após paralisia cerebral, acidente vascular cerebral, **DP**, ataxia cerebelar, traumatismo cranioencefálico e paralisia supranuclear. **83%** dos estudos encontraram **efeitos positivos do NIBS.** A diferença significativa após-tratamento entre os grupos sham e tratamento foi associada apenas com a **taxa de movimento sequencial** (Hedges 'g = 0,79, 95% CI [0,075, 1,50] P = 0,03) **favorecendo a tDCS.**	A maioria dos estudos que identificaram os benefícios da SBNI não forneceu evidências para a recuperação completa da disartria. Investigações clínicas em larga escala são necessárias para avaliar a eficácia da SBNI no tratamento da disartria.
BALZANA, P et al (2022)	Annals of Physical and Rehabilitation Medicine DOI: 10.1016/j.rehab.2021.101580	RS: Estimulação cerebral não invasiva para o tratamento da disartria neurogênica.	Examinar a base de evidências de estimulação central e periférica não invasiva para melhorar as funções relacionadas à fala em pessoas com disartria e determinar os efeitos de uma intervenção	**BASES DE DADOS:** MEDLINE PsycINFO EMBASE CINAHL Linguistics and Language Behavior Abstracts Web of Science O Registro Cochrane de Ensaios de Controle e 2 registros de ensaios foram concluídos **PALAVRAS CHAVES:** Estimulação cerebral e elétrica, Disartria e Projeto de pesquisa.	Foram identificados 6.186 estudos; 10 estudos (6 ensaios clínicos randomizados e 4 estudos cruzados) preencheram os critérios de inclusão. Os 10 estudos (268 adultos com doença de Parkinson, acidente vascular cerebral e ataxia cerebelar neurodegenerativa) focaram na NIBS (6 TMS; 3 tDCS; e 1 tACS) . A maioria dos ensaios relatou um ou mais efeitos positivos da estimulação nas características relacionadas à disartria. Os 3 estudos forneceram estimulação anódica sobre o cerebelo ou M1, com uma intensidade de corrente de 2mA. A tDCS foi administrada por 20(n=2) ou 30 (n=1)min. Em todos os ensaios, a estimulação foi administrada 5 vezes por semana para o total de 10 sessões.	As evidências do uso de estimulação cerebral não invasiva no tratamento da disartria permanecem inconclusivas. São necessários ensaios de investigação que forneçam resultados fiáveis e replicáveis.

**Legenda: tDCS -** Transcranial direct current stimulation **(**Estimulação Transcraniana por Corrente Contínua) **atDCS -** Estimulação Transcraniana por Corrente Contínua Anódica **ctDCS -** Estimulação Transcraniana por Corrente Contínua Catódica **sham ou stDCS -** Estimulação Transcraniana por Corrente Contínua simulada **DP -** Doença de Parkinson **AVC** - Acidente Vascular Cerebral **DBS -** Deep Brain Stimulation (Estimulação Cerebral Profunda) **EAV** - Escala Analógica Visual **NHR** - Proporção Harmônico Ruído **AMS** - Área Suplementar Motora **UPDRS** - Escala Unificada de Avaliação da Doença de Parkinson **DLPFC**- Córtex Pré-Frontal Dorsolateral **TPC** - Córtex Temporoparietal Esquerdo **FP2**- Córtex Órbito Frontal **LSVT** - Lee Silverman Voice Treatment **MoCA** - Avaliação Cognitiva Montreal.

**Tabela 3:** Descrição de artigos empíricos incluídos na Metanálise sobre a Estimulação Transcraniana por Corrente Contínua (tDCS) para o tratamento de voz ou fala na DP.

**AUTORES/ANO**	**REVISTA / DOI DA PUBLICAÇÃO**	**TIPO DE ESTUDO / TÉCNICA DE TRATAMENTO**	**OBJETIVO**	**MÉTODO / PARÂMETROS DE tDCS**	**PRINCIPAIS ACHADOS**	**CONCLUSÃO**
PEREIRA, JB et al (2013)	Brain Stimulation **DOI:** 10.1016/j.brs.2012.01.006	**C. EMPÍRICOS** Ensaio Clínico tDCS Modulação de redes de fluência verbal por estimulação transcraniana por corrente contínua (tDCS) na doença de Parkinson.	Avaliar os efeitos da tDCS nas redes funcionais da fluência fonêmica e semântica em pacientes com DP.	- 16 pacientes com DP idiopática; N=16 - Escala Unificada de Avaliação da Doença de Parkinson (UPDRS) e Escala Hoehn & Yahr; Mini mental e Escala de Depressão; - Testes Neuropsicológicos. tDCS em 2mA por 20 min; atDCS sobre F3(DLPFC esq) ou P3-T5 (TPC esq) e ctDCS sobre FP2 dir.	-Efeitos da **tDCS na fluência verbal** (F (1,15) = 14,079, P<0,002), A estimulação do DLPFC aumentou a quantidade de palavras produzidas. -Efeitos da **tDCS na fluência semântica**- F(1,15) = 3,092, P<0,102) A estimulação do DLPFC aumentou a quantidade de palavras produzidas.	A tDCS melhorou mais a conectividade funcional na fluência verbal e nas redes relacionadas a tarefas de desativação quando aplicada sobre o DLPFC do que o TPC na DP.
PIETROBON et al. (2021)	Audiology Communication Research **DOI:** 10.1590/2317-6431-2020-2343	Caso Clínico tDCS Terapia de produção audiovisual associada à estimulação por corrente transcraniana melhora nomeação em paciente com afasia de Broca e doença de Parkinson	Estimular a área de Broca com tDCS decorrente ao déficit em tarefas de nomeação, no córtex pré-frontal inferior esquerdo (área de Broca).	-Um paciente de 79 anos com DP (estágio 4 na escala de Hoehn e Yahr) e afasia crônica não fluente pós-AVC, com déficit de nomeação severos; N=1 - Combinação de terapia audiovisual de produção e tDCS anódica (2 mA) aplicada sobre o córtex pré frontal inferior esquerdo (F7), nove sessões de 20 min + nomeação de imagens de objetos comuns com o auxílio de vídeos curtos.	Aumento significativo nos escores de nomeação entre o pré e o pós tratamento, tanto para imagens treinadas ou não treinadas, mas foneticamente similares (generalização).	Os resultados apresentaram indícios iniciais que a terapia audiovisual de produção associada à tDCS anódica sobre a área de Broca pode representar uma alternativa viável para pacientes com déficits de nomeação severos.
LEYDON,C et al. (2022)	Perspectives - SIG 2 Neurogenic Communication Disorders **DOI:** 10.1044/2021_PERSP-21-00040	Ensaio Clínico tDCS Alterações na fala após estimulação transcraniana por corrente contínua em indivíduos com diagnóstico de doença de Parkinson	Examinar o impacto da tDCS na produção da fala em indivíduos diagnosticados com DP	-Seis homens, entre 65 e 78 anos, com diagnóstico de DP. N=6 -Estudo duplo-cego cruzado controlado para os efeitos imediatos da tDCS no córtex pré-motor para a produção da fala. - - Auditiva- perceptivo (EAV) e resultados acústicos da voz, (frequência fundamental, intensidade, NHR e velocidade de fala) pré e pós-simulação anodal e sham.	- Velocidade de fala abaixo dos níveis normativos para todos os participantes antes da tDCS. A taxa de discurso aumentou significativamente na conversação após a estimulação, mas não na tDCS sham, excedeu os valores normativos para dois participantes. O NHR aumentou após a estimulação. Não houve diferença significativa em outras medidas de perturbação, classificações perceptuais, fundamentais frequência ou intensidade foram anotadas.	Todos os participantes completaram o protocolo sem desconforto. O aumento observado na velocidade de fala precisa ser interpretado no contexto de excitação resultante da neuroestimulação com tDCS.
ROSA, RR, et al. (2023)	Audiology Communication Research **DOI:** 10.1590/2317-6431-2023-2795pt	Caso Clínico tDCS Efeitos da estimulação transcraniana por corrente contínua (tDCS) na voz e fala na doença de Parkinson: relato de caso	Comparar a efetividade da terapia fonoaudiológica convencional combinada com tDCS e tDCS isoladamente.	-Dois pacientes do sexo masculino com DP e disartria hipocinética que foram submetidos à avaliação fonoaudiológica. N=2 S1- 10 sessões de 20 minutos de tDCS S 2 - 10 sessões de 20 minutos de tDCS + terapia fonoaudiológica Avaliados pré e pós com follow up de 30 dias. 10 dias consecutivos de tDCS anódica em córtex motor primário (M1), Sistema 10-20 (C3) e catódica (FP2)	-A melhora no tempo de fonação, movimento velar e outras medidas de disartria foram mais significativas no S1. -A análise acústica de fonte glótica mostrou melhora em medidas de frequência e Jitter, Shimmer e ruído maior S2. -S1 apresentou melhores resultados na avaliação perceptivo- auditiva de fala e voz, enquanto S2 obteve melhores escores na análise acústica.	A terapia fonoaudiológica convencional associada à tDCS apresenta impacto mais significativo na fala e voz do que a tDCS isolada, demonstrando o potencial da tDCS como tratamento complementar para pacientes com DP.
JOHARI K (2024)	Cochrane Library - Cochrane Registro Central de Ensaios Controlados	Ensaio Clínico tDCS Resultados de fala e voz após HD-tDCS sobre o AMS esquerda	Investigar os efeitos imediatos e de curto prazo da HD-tDCS na área motora suplementar esquerda (AMS) sobre os déficits de fala e voz	24 participantes com DP e 24 controles pareados. N=48 tDCS anódica sobre Área Motora Suplementar(AMS) 2mA / 20 minutos 5 dias consecutivos Realização de protocolo de ensaio clínico randomizado registrado na Cochrane acerca dos efeitos imediatos e de curto prazo da estimulação transcraniana por corrente contínua de alta definição (tDCS de HD) sobre a área motora suplementar esquerda (AMS) sobre os déficits de fala e voz.	Evidências crescentes apoiam a aplicação de técnicas de estimulação cerebral não invasivas, como a tDCS para melhorar os sintomas motores e não motores na DP. No entanto, existem evidências limitadas sobre a aplicação da tDCS na melhora dos distúrbios de fala e voz na DP. Além disso, não há efeito estabelecido a longo prazo da tDCS sobre os déficits de fala e voz na DP.	Ainda não houveram resultados, o estudo está em andamento.
						
BRABENEC, L et al (2024)	Journal of Neural Transmission **DOI:** 10.1007/s00702-024-02771-5	Ensaio Clínico tDCS Efeitos de curto prazo da estimulação transcraniana por corrente contínua na fala motora na doença de Parkinson: um estudo piloto	Desenvolver um programa para tratamento remoto de HD-tDCS combinada com o LSVT^®^	-14 pacientes destros com DP clinicamente estabelecida. N=14 - (MoCA) para demência, MoCA>20 ; medicação ON sem discinesias; presença de sintomas de Disartria -Três sessões de estimulação tDCS (estimulação anódica, catódica e simulada) de 2 mA por 20 min, separadas por um dia com tarefas de fala que duraram até 10 minutos com 150 palavras.	Pela análise de modelo linear misto (LMM) observou-se um efeito significativo da condição de estimulação nas mudanças na duração mediana de silêncios superiores a 50 ms (F(2,21,8)=5,1, p=0,015 ), silêncios inadequados que impactam negativamente o ritmo e a fluência da fala.	A tDCS anódica visando a área de feedback auditivo no hemisfério direito pode melhorar significativamente a fluência motora da fala em pacientes com DP.
						

**Legenda: tDCS -** Transcranial direct current stimulation **(**Estimulação Transcraniana por Corrente Contínua) **atDCS -** Estimulação Transcraniana por Corrente Contínua Anódica **ctDCS -** Estimulação Transcraniana por Corrente Contínua Catódica **sham ou stDCS -** Estimulação Transcraniana por Corrente Contínua simulada **DP -** Doença de Parkinson **AVC** - Acidente Vascular Cerebral **DBS -** Deep Brain Stimulation (Estimulação Cerebral Profunda) **EAV** - Escala Analógica Visual **NHR** - Proporção Harmônico Ruído **AMS** - Área Suplementar Motora **UPDRS** - Escala Unificada de Avaliação da Doença de Parkinson **DLPFC**- Córtex Pré-Frontal Dorsolateral **TPC** - Córtex Temporoparietal Esquerdo **FP2**- Córtex Órbito Frontal **LSVT** - Lee Silverman Voice Treatment **MoCA** - Avaliação Cognitiva Montreal.

O Diagrama PRISMA (2020) com as fases de Identificação, Seleção e Inclusão dos artigos desta Revisão Sistemática e Metanálise está apresentado na Figura 1 abaixo.

**Figura 1:** Fluxograma PRISMA (2020) - visão geral do processo de triagem e exclusão dos diferentes artigos

### Descrição dos estudos

Os estudos incluídos nesta Revisão Sistemática foram descritos por tópicos em forma de tabela e serão discutidos com outros estudos e achados da literatura sobre o tema da pergunta de pesquisa.

#### Revisão sistemática

As Tabela 2 apresenta as oito publicações selecionadas para esta revisão que se referem às publicações teóricas, conceituais ou de Revisão Sistemática que não tinham dados empíricos para a inclusão na Metanálise, e que utilizaram a Estimulação Transcraniana por Corrente Contínua (tDCS) para a DP, mostrando a identificação dos autores e ano de publicação, local da publicação, DOI, objetivos, o método utilizado, parâmetros de tDCS, principais achados e a conclusão do estudo.

#### Metanálise

As publicações de estudos empíricos com os seis artigos que compõem esta Metanálise estão descritas na Tabela 3 com os mesmos tópicos já indicados na Tabela 2 para a utilização da tDCS para fala e voz na DP.

E a Tabela 4 aponta as seis publicações de artigos empíricos incluídas na Metanálise que se referem a utilização da Estimulação Transcraniana por Corrente Contínua (tDCS) para o tratamento da doença de Parkinson com desfecho para voz e fala, que serão descritos em seguida. A tabela descreve o tipo de estimulação de neuromodulação, o status da medicação durante a sessão, os locais de estimulação (ânodo/cátodo/sham), tamanho dos eletrodos, intensidade da corrente(mA), duração em minutos da estimulação e número de sessões.

**Tabela 4:** Parâmetros de tDCS dos artigos incluídos na Revisão Sistemática com Metanálise de tDCS para o tratamento de voz e/ou fala em DP.

**AUTOR/ANO** **APARELHO DE tDCS**	**TIPO DE ESTIMULAÇÃO**	**STATUS DA MEDICAÇÃO**	**LOCAL DE ESTIMULAÇÃO** **(ÂNODO/CÁTODO)**	**TAMANHO DO ELETRODO**	**INTENSIDADE** **(mA)**	**DURAÇÃO EM MINUTOS**	**NÚMERO DE SESSÕES**
PEREIRA, JB et al (2013) Phoresor, Iomed Inc.(EUA)	Experimento cruzado de tDCS combinado com fMRI	ON	**ÂNODO** **F3**(DLPFC)ESQUERDO OU **P3-T5**(TPC)ESQUERDO **CÁTODO** **FP2** DIREITO	**UM PAR DE ELETRODOS** ESPONJA 35 cm2	2mA	20 MINUTOS	1 SESSÃO (intervalo- 2h entre os experimentos)
PIETROBON et al. (2021) DC-STIMULATOR, neuroConn (Alemanha)	Combinação de terapia audiovisual de produção verbal e tDCS anódica	ON	**ÂNODO** **F7** HEMISFÉRIO ESQUERDO(BROCA) **CÁTODO** **FP2** DIREITO	**DOIS ELETRODOS** ESPONJA 35 cm2	2mA	20 MINUTOS	9 SESSÕES
LEYDON,C et al. (2022) Neuroelectrics Starstim (Suíça)	Estudo cruzado dos efeitos imediatos da tDCS no córtex pré-motor da produção de fala	ON	**ÂNODO** **F3**(DLPFC)ESQUERDO **SHAM** **F3**(DLPFC)ESQUERDO	**ELETRODOS** Ag/AgCl	2mA	20 MINUTOS	2 SESSÕES (cruzadas - ativa ou sham com intervalo de 1-2 semanas)
ROSA, RR, et al. (2023) NKL Microestim Foco tDCS (Brasil)	Efeitos da tDCS na voz e fala na doença de Parkinson	ON	**ÂNODO** **C3 (**M1 - CÓRTEX MOTOR PRIMÁRIO**)** **CÁTODO** **FP2** DIREITO .	**DOIS ELETRODOS** ESPONJA 7×5 cm2	2mA	20 MINUTOS	10 SESSÕES
JOHARI K (2024) Soterix HD-tES de 9 MxN. (New York)	Resultados de fala e voz após HD-tDCS sobre o SMA esquerdo	ON	**ÂNODO** **F3**(DLPFC)ESQUERDO **CÁTODO** **FP2** DIREITO .	**ELETRODOS** **HD** (diâmetro de 1,2 cm) com tampa HD-tDCS	2mA	20 MINUTOS	5 SESSÕES CONSECUTIVAS
BRABENEC, L et al (2024) DC-STIMULATOR PLUS (Alemanha)	Efeitos de curto prazo da tDCS na fala motora da doença de Parkinson	ON	**ANÓDICA** ÂNODO- STG POSTERIOR DIREITO CÁTODO- STG POSTERIOR ESQUERDO **CATÓDICA** CÁTODO - STG POSTERIOR DIREITO ÂNODO - STG POSTERIOR ESQUERDO	**DOIS ELETRODOS** ESPONJA 5×5 cm2	2mA	20 MINUTOS	3 SESSÕES **ANÓDICA** **CATÓDICA** **SHAM** SEPARADAS POR UM DIA SEM ESTIMULAÇÃO
							

**Legenda das siglas: tDCS -** Transcranial direct current stimulation **(**Estimulação Transcraniana por Corrente Contínua) **fMRI -** Ressonância Magnética Funcional **TPC** - Córtex Temporoparietal Esquerdo **FP2**- Córtex Orbitofrontal **DLPFC**- Córtex Pré-Frontal Dorsolateral **CPF** - Córtex Pré-Frontal **TC** - Córtex Temporal **M1** - Córtex Motor primário **IPFC -** Córtex Pré-Frontal Inferior **STG** - Giro Temporal Superior **mA-** microampere.

### Análise dos resultados

Para esse estudo de Metanálise os resultados foram comparados quanto ao desfecho em comum dos estudos para fala e voz com a aplicação da tDCS anódica ou catódica em comparação com o sham ou entre elas mesmas. Foi utilizada como referência a média entre o grupo que recebeu a estimulação e o que não recebeu. Utilizamos o software Review Manager 5.4.1 para calcular o efeito do tratamento e os intervalos de confiança(95%) dos estudos, utilizando a média, o desvio padrão e o tamanho da amostra, analisando os resultados e forest plot. Observamos também o risco de viés para os estudos incluídos nesta metanálise: viés de seleção (randomização e alocação), viés de desempenho (blindagem de participantes e pessoal), viés de detecção (blindagem da avaliação dos resultados), viés de atrito (resultados incompletos), viés de relatório (relatórios seletivos) e outros, sendo classificados quanto ao risco de viés em: baixo risco, risco pouco claro ou alto risco.

A Metanálise realizada com os artigos incluídos será apresentada de acordo com a área alvo para estimulação com tDCS. Podemos observar na Figura 2 o efeito do tratamento e os intervalos de confiança(95%) dos estudos, pela tabela e forest plot dos estudos com tamanho de efeito geral de Z= 0.89(P=0.37), e significância de (P<0.00001) para heterogeneidade, apresentado pelo modelo de efeitos aleatórios.

**Figura 2:** Metanálise dos estudos de tDCS para o tratamento de voz e/ou fala em DP com as áreas alvo para Córtex Pré-Frontal (FPC), Córtex Motor (M1) e Córtex Temporal Superior (STC)

**Legenda das siglas: tDCS -** Transcranial direct current stimulation **(**Estimulação Transcraniana por Corrente Contínua) **PFC -** Córtex Pré-Frontal **M1** - Córtex Motor primário **STC** - Córtex Temporal Superior

O *forest plot* mostrou para a área alvo do córtex pré-frontal (FPC) três estudos: Leydon et al.^(25)^, Pereira et al.^(24)^ e Pietrobon et al.^(21)^; com tamanho de efeito geral de 1.38(P=0.17), com significância de (P<0.00001) para heterogeneidade; no alvo do córtex motor primário(M1) um estudo (Rosa, et al. 2023) com tamanho de efeito de 0.23(P=0.82); e na área do córtex temporal superior(STC) dois estudos: Brabenec et al. (2023) e Pereira, et al. (2013) com tamanho de efeito 0.89(P=0.86) e significância de (P<0.00001) para heterogeneidade. Nestes resultados observamos quanto maior o tamanho do efeito, ou força da associação, mais impacto o fator em estudo tem no desfecho, trazendo os estudos com área alvo do FPC com maior tamanho de efeito, portanto com maior impacto para desfecho de fala e voz com uso de tDCS para DP.

Foi observada uma alta heterogeneidade na meta-análise em decorrência possivelmente das diferenças nos protocolos de intervenção com relação a localização da aplicação da tDCS e a duração do tratamento. A análise dos subgrupos nos mostra que a estimulação de tDCS com área alvo do córtex pré-frontal (FPC) obteve maior tamanho de efeito por corresponder a área cortical funcional responsável pelo controle motor da produção da voz, através da via cortical motora laríngea, com o córtex pré frontal dorsolateral (DLFPC) responsável pelas tarefas aprendidas de falar e cantar, regulando o controle da produção e da frequência da voz e; o córtex pré frontal ventromedial (VMFPC) com o centro laríngeo da deglutição para proteção das vias aéreas^(9)^.

Na avaliação do risco de viés por meio da ferramenta RoB 2.0, observamos na avaliação qualitativa *baixo risco de viés* para a maioria dos estudos nos domínios randomização, alocação, resultados e relatórios (Leydon et al., 2022; Pereira et al, 2013 e Brabenec et al., 2023), no domínio de blindagem dos participantes e pessoal a avaliação qualitativa foi de *risco pouco claro* para os seguintes estudos: Pereira et al. (2013), Pietrobon et al. (2021) e Rosa et al. (2023). E para o domínio de randomização e alocação também apresentou *risco pouco claro* para o estudo de Rosa et al. (2023) por não se aplicar.

E por fim, foi utilizado o GRADEpro, versão on-line, uma ferramenta sistemática para avaliar a certeza da evidência e a força das recomendações clínicas. Após a análise dos resultados pelos tópicos descritos no GRADE, pode-se classificar os estudos incluídos nesta Metanálise pelo GRADE como ***Baixa*** certeza de evidência, como observado na Figura 3, que descreve os itens da avaliação pelo GRADE para este estudo.

**Figura 3:** GRADEpro dos estudos de tDCS para o tratamento de voz e/ou fala em DP para avaliar a certeza da evidência e a força das recomendações clínicas

Quanto ao *risco de viés* e a *evidência indireta*, foram classificados como *não grave*; na *inconsistência* classificados como *grave*, baixou-se um nível na inconsistência devido ao valor da heterogeneidade (>75%), possivelmente pelos protocolos de intervenção diferentes e follow-ups distintos nos estudos, e na *imprecisão* (grave), justifica-se baixar um nível de evidência porque os estudos mostraram benefício, mas não foram significativos. Pode-se concluir que é muito provável que pesquisas futuras tragam um impacto importante na confiança da estimativa do efeito e podem alterar essa estimativa.

A utilização deste recurso metodológico garante que as decisões baseadas em evidência sejam tão confiáveis quanto possível, promovendo melhores resultados de saúde.

## DISCUSSÃO

Esta seção traz conceitos da Neuromodulação não-invasiva pela tDCS e como este recurso terapêutico vem impactando na reabilitação dos distúrbios e alterações neurológicas, mais especificamente na Doença de Parkinson. Os artigos e publicações teóricas e conceituais nos trazem nesta RS informações relevantes e atuais sobre o tema.

### Revisão sistemática

Inicialmente, um estudo realizado por Fox et al. (2014) fez um levantamento sobre a estimulação cerebral invasiva e não-invasiva em doenças psiquiátricas e neurológicas, mostrando os locais e áreas alvo mais eficazes para cada doença, de acordo com a conectividade funcional em estado de repouso. Podemos observar entre elas algumas das áreas estimuladas: Doença de Alzheimer (TMS ou ETCC - DLPFC bilateral + parietal/temporal),Depressão(TMS ou ETCC - DLPFCe/ DLPFCd), Dor(TMS ou ETCC - M1), e Doença de Parkinson - TMS ou ETCC(M1, SMA) entre outros^(10)^.

Dois estudos conduzidos por Miterko et al.^(11)^, Manto et al.^(12)^, relataram em forma de consenso e artigo conceitual a utilização da Neuroestimulação no Cerebelo para a DP, com as afirmações que as comunicações anatômicas entre o cerebelo e os gânglios da base proporcionam ampliação da aplicação e resultados da tDCS para as distonias e outras doenças neurológicas.

A tDCS anódica cerebelar melhora os resultados de tarefas de caligrafia, desenhos em círculos e reabilita a fala e déficits de linguagem. Apresentaram resultados positivos também para a fluência verbal e sintomas cognitivos na DP, além de aumentar a capacidade de aprender com erros, formar e reter a memória do ato motor. Manto(2022) observou que pela neuroimagem funcional a fluência verbal recebe influência da neuroestimulação cerebelar, principalmente a do tipo fonêmica pela tDCS cerebelar anódica na região póstero-lateral direita. Verificou também que houve percepção de mudança do tom da voz de vogais sustentadas comparados ao sham, quando 19 pacientes receberam tDCS cerebelar com neuronavegação, promovendo menores compensações vocais para perturbação de pitch, confirmando que a tDCS cerebelar pode modular a integração auditivo-vocal alterada^(11,12)^.

A aplicação de estimulação contínua theta-burst (cTBS) e de tDCS cerebelar têm mostrado sua eficácia e efeitos positivos por outros autores com benefícios para os aspectos de fala e voz. Segundo autores^(13)^ a ativação anódica por tDCS do cerebelo direito foi observada através de tarefas de geração verbal, memória de trabalho verbal, produção de voz e ajuste compensatório da voz. Além desses aspectos observados e ainda mais importante é a relação causal encontrada nos estudos entre o cerebelo direito e a produção de fala, mostrando pelos resultados dos autores que a tDCS sob o cerebelo direito resultou em compensações vocais aumentadas para perturbações de pitch ou de perturbações de F1.

Estudos têm evidenciado também que a utilização da tDCS cerebelar anódica antes ou durante as tarefas de fala e produção de voz leva a compensações vocais aprimoradas para perturbações de pitch, com magnitude e latência de respostas vocais não moduladas pelo tempo de estimulação. Além disso, os efeitos modulatórios da tDCS cerebelar anódica nas compensações vocais foram observados independentemente do tamanho e direção das perturbações de pitch^(14)^.

Outros autores, Montemurro et al.^(15)^, Saluja et al. ^(16)^ e Weismer et al.^(17)^ et al realizaram revisões de literatura sobre a tDCS e relataram evidências atuais sobre a aplicação da Neuromodulação em pacientes com DP com relação a voz e fala. Observaram que a tDCS favorece os resultados nos sintomas motores da DP de forma mais proeminente quando relaciona à reabilitação, provavelmente pela plasticidade neuronal e geração de sinapses de longo prazo (BNDF).

No que se refere a reabilitação de fala e linguagem em DP, consideram que cerca de 89% dos pacientes com DP podem desenvolver *distúrbios de fala e voz*(redução de volume, voz monótona e qualidade soprosa), *articulação*(consoantes imprecisas e centralização vocálica) e *frequência* (aumentada, diminuída e variável). A terapia fonoaudiológica com tDCS melhorou os níveis de pressão sonora na vogal sustentada, apresentou redução da pontuação do índice de desvantagem vocal e melhorou a leitura de texto e monólogo. Observou-se também que a tDCS aplicada aos movimentos da mandíbula associados à fala, repetição de sílabas e mastigação reforçou o conceito de que os efeitos da tDCS dependem da tarefa, já que apresentou uma relação negativa entre o desempenho oromotor não-verbal e as medidas de produção de fala^(15-17)^.

Além da área alvo do cerebelo para regulação de pitch vocal, muitos estudos da Neuromodulação em tDCS estão em foco no feedback auditivo para o controle motor da fala, pois esse aspecto fornece informações sensoriais que permitem o monitoramento e ajuste da saída vocal a fim de produzir os objetivos de fala pretendidos. Esse processo de controle é conhecido como interação auditivo-motora, manifestada por ajuste compensatório em resposta a incompatibilidades entre o feedback auditivo esperado e o produzido na frequência fundamental(F0), na intensidade ou no primeiro formante(F1) da voz^(18)^.

Esses autores^(18)^ em sua pesquisa utilizaram a tDCS anódica em 17 adultos saudáveis para modular a excitabilidade cortical do DLPFC esquerdo e examinar seus efeitos na integração auditivo-motora para regulação do tom vocal, os participantes vocalizaram sons de vogais enquanto ouviam sua voz pseudo-aleatoriamente alterada quanto a frequência para maior/menor hz, durante ou após a tDCS anódica ativa ou simulada(sham) sob o DLPFC esquerdo. Nos resultados, a tDCS anódica ativa sobre o DLPFC esquerdo levou a magnitudes de pico significativamente menores e tempos de pico mais curtos de compensações vocais para perturbações de tom do que a estimulação simulada.

E por fim as duas revisões sistemáticas de Baig et al.^(19)^ e Balzana et al.^(20)^ que utilizaram a busca nas bases de dados para verificar as evidências sobre a utilidade da Neuromodulação não-invasiva pela tDCS no tratamento da disartria. As bases de dados pesquisadas e palavras-chaves para a RS de Baig (2022) foram: Google Scholar, Embase, Scopus, PubMed, Cochrane e Web of Science, palavras-chave(Disartria, tDCS, EMT, Distúrbio neurológico), com 9 estudos com TMS e 5 com tDCS. A terapia fonoaudiológica associada a tDCS foi oferecida em 6 estudos, em 83% dos estudos foram encontrados efeitos positivos da tDCS. A diferença significativa pós-tratamento entre os grupos sham e tratamento foi associada com a taxa de movimento sequencial (Hedges'g = 0,79, 95% CI [0,075, 1,50] P = 0,03) favorecendo a tDCS^(19)^.

E a RS de Balzana et al.^(20)^ teve as seguintes buscas: Medline, PsycInfo, Embase, Linguistics and Language Behavior Abstracts, Web of Science e Cochrane, palavras-chave (Estimulação cerebral e elétrica, Disartria e Projeto de pesquisa), encontrando 10 estudos (6 ensaios clínicos randomizados e 4 estudos cruzados) focados na Neuromodulação(6 de TMS, 3 de tDCS e 1 de tACS) em 268 adultos com DP, AVC e ataxia cerebelar neurodegenerativa, relacionadas à disartria. Os 3 estudos forneceram estimulação anódica sobre o cerebelo ou M1,com uma intensidade de corrente de 2mA. A tDCS foi administrada por 20(n=2) ou 30(n=1) minutos. Em todos os ensaios, a estimulação foi administrada 5 vezes por semana para o total de 10 sessões, e mostrou que as evidências do uso da tDCS no tratamento da disartria precisam de mais ensaios clínicos randomizados para que seus resultados sejam replicados com mais eficácia^(20)^.

### Meta-análise

Nesta revisão foram selecionados também seis artigos empíricos, que foram incluídos para a Meta-análise, quatro ensaios clínicos e dois de relatos de casos clínicos no que se refere aos sintomas de fala e voz apresentados antes e após a aplicação da tDCS para DP nas áreas alvo do córtex pré-frontal (FPC), córtex motor (M1) e córtex temporal superior(STC).

Pietrobon et al.^(21)^ e Rosa et al.^(22)^ realizaram estudos de casos clínicos com os seguintes métodos e resultados. Pietrobon et al. (2021) estimulou com tDCS anódica, 2mA por 20 minutos, em 9 sessões, a área de Broca, no córtex pré-frontal inferior esquerdo,(F7) pelo sistema 10-20, em um paciente de 79 anos com DP e afasia crônica não-fluente pós AVC e déficit de nomeação severo, associado a terapia audiovisual de nomeação de imagens de objetos comuns com auxílio de vídeos. Como resultados obtiveram aumento significativo nos escores de nomeação entre o pré e o pós tratamento^(21)^.

E Rosa et al. (2023) verificou os efeitos da tDCS na voz e na fala na DP, com o estudo de dois pacientes do sexo masculino com DP e disartria hipocinética, com a aplicação de 10 sessões de tDCS anódica em córtex motor primário (M1), pelo sistema 10-20(C3) e tDCS catódica em (FP2),com intensidade de 2mA, pelo tempo de 20 minutos, em 10 dias consecutivos. Um paciente recebeu terapia fonoaudiológica concomitante a neuromodulação e o outro não recebeu. Em seus resultados, os autores perceberam melhora no tempo de fonação, no movimento velar e parâmetros de análise acústica (medidas de frequência, jitter, shimmer e ruído)para os participantes e concluíram que a terapia fonoaudiológica convencional associada à tDCS apresenta melhores resultados na fala e voz do que a tDCS aplicada de forma isolada^(22)^.

Johari et al. (2024) ainda não apresentou resultados em seu estudo, após leitura completa observou-se que era um registro de protocolo de ensaio clínico para aplicação de HD-tDCS sobre a área motora suplementar esquerda (AMS), onde o objetivo é investigar os efeitos imediatos e de curto prazo da HD-tDCS sobre os déficits de fala e voz. A pretensão é de parear 24 participantes com DP e 24 controles com a utilização de HD-tDCS anódica com 2mA, por 20 minutos, em 5 dias consecutivos na AMS esquerda. Este é um protocolo de ensaio clínico randomizado registrado na Cochrane com início previsto para o ano de 2024^(23)^.

E os três com ensaios clínicos randomizados foram os dos autores: Pereira et al.^(24)^, Leydon et al.^(25)^ e Brabenec et al.^(26)^ com aplicação de tDCS em áreas cerebrais para fala e voz em DP. Pereira et al(2013) verificaram a modulação de redes de fluência verbal por tDCS na Doença de Parkinson, eles avaliaram os efeitos da tDCS em redes funcionais da fluência fonêmica e semântica em 16 pacientes com DP idiopática, aplicaram escalas de avaliação, cognitiva e de depressão, seguidas da aplicação da tDCS anódica sobre o DLPFC ou TPC esquerdo, e catódica sobre o FP2 direito, com intensidade de 2mA, por 20 minutos, em uma sessão com intervalo de 2 horas entre os experimentos. Verificaram que a tDCS melhorou a conectividade funcional na fluência verbal quando aplicada sobre o DLPFC mais do que sobre o TPC na DP^(24)^.

Outros autores, Leydon et al.^(25)^ observaram alterações na fala em indivíduos com diagnóstico de DP e avaliaram o impacto da aplicação da tDCS. O estudo foi composto por 6 homens com DP, idade entre 65 e 78 anos, com desenho de estudo duplo cego, cruzado e controlado, para os efeitos imediatos da tDCS no córtex pré-motor na produção da fala. Foi estimulado com 2mA, por 20 minutos, o DLPFC esquerdo com tDCS anódica e sham cruzada, com intervalo de uma/duas semanas na estimulação. Foram avaliados os seguintes parâmetros antes e após a estimulação ativa e simulada: avaliação perceptivo auditiva(EAV) e resultados acústicos da voz(frequência fundamental, intensidade, NHR e velocidade de fala). Nos resultados foi encontrada velocidade de fala abaixo da normalidade para os participantes antes da tDCS e após a neuromodulação houve um aumento da taxa de discurso significativa na conversação após a estimulação, o que não ocorreu na tDCS simulada(sham). O NHR aumentou após a estimulação, porém as outras medidas de perturbação perceptuais, frequência fundamental ou intensidade não apresentaram diferença estatisticamente significativa.

E por fim, o estudo de Brabenec et al(2024) que analisou os efeitos de curto prazo da HD-tDCS na fala motora em DP, em estudo piloto de tratamento remoto combinado ao LSVT^®^, na população da pesquisa foram 14 pacientes com DP diagnosticada, em estado ON da medicação e presença de sintomas de disartria. Foram aplicadas três sessões de tDCS(anódica, catódica e simulada), com a intensidade de 2mA por 20 minutos, separadas por um dia, e aplicação de tarefas de fala com 150 palavras. Os resultados mostraram efeito significativo da estimulação na melhora da duração mediana dos silêncios inadequados no discurso, que quando alterados impactam negativamente o ritmo e a fluência de fala. Os autores concluíram que a tDCS anódica visando o feedback auditivo pode melhorar a fluência motora de fala de indivíduos com DP^(26)^.

Os resultados foram descritos pelos estudos conceituais e de revisão de literatura/sistemática nesta RS trazendo os benefícios e eficácia da aplicação da tDCS. E no que se refere aos estudos empíricos, na sua maioria apresentou-se com a aplicação da tDCS do tipo anódica sob áreas específicas do cérebro, com intensidade de 2mA por 20 minutos, com maior efeito para a aplicação no córtex pré-frontal, como verificado na Meta-análise, para os efeitos e evidências de voz e fala, com melhora nos níveis de pressão sonora da vogal sustentada, melhora na fluência verbal (fonêmica), leitura de palavras e monólogo.

### Limitações e perspectivas do estudo

Entre as limitações encontradas nesta Meta-análise apresentam-se ainda algumas lacunas de conhecimento a serem esclarecidas quanto aos protocolos utilizados: quantidade e duração da estimulação e áreas alvo mais adequadas para potencializar resultados para voz e fala com a utilização de tDCS em DP.

Atualmente existe uma baixa quantidade de estudos nesta área pesquisada, e podemos observar a presença de possíveis vieses nos estudos, pela heterogeneidade encontrada na meta-análise, o que mostra a necessidade da realização de estudos mais robustos e bem controlados a fim de comprovar os benefícios e resultados já encontrados na literatura científica, como também na prática clínica para a população em estudo.

Torna-se necessário promover a realização de estudos maiores e ensaios clínicos randomizados com melhores delineamentos metodológicos que possam investigar com mais precisão os resultados e a duração dos efeitos da tDCS associada a recursos terapêuticos de voz e fala para a DP.

## CONCLUSÃO

Após observar o que foi exposto nesta RS com Metanálise sobre a utilização da tDCS nas alterações de fala e voz nos indivíduos com Parkinson, percebe-se que a tDCS é um recurso terapêutico que vem sendo implementado no tratamento da DP há alguns anos, de forma complementar às terapias e como alternativa a outras modalidades de terapia invasiva ou não-invasiva para Parkinson, tais como a cirurgia de Deep Brain Stimulation(DBS) ou a utilização da Transcranial Magnetic Stimulation(TMS), entre outras.

Os estudos da Metanálise com áreas alvo do córtex pré-frontal, área motora primária e temporal superior foram os que mostraram melhores evidências e impacto para o desfecho de fala e voz com uso de tDCS para a DP, principalmente por proporcionar regulação pelo feedback auditivo no ato motor da fala e voz, regulando parâmetros como pressão sonora, frequência fundamental (F0) e primeiro formante (F1), além de fluência verbal e leitura de palavras. Conclui-se que as áreas alvo estimuladas com a tDCS podem trazer benefícios através da conectividade funcional e conexões estabelecidas para a produção e regulação da fala e voz na DP.

A realização da RS e da Metanálise nesta temática são encorajadoras e estabelecem modelos que podem proporcionar o desenvolvimento de ensaios clínicos que visam respaldar a aplicação da tDCS no tratamento de fala e voz da DP, e que com base em evidência científica se consolida como uma opção terapêutica viável na fonoterapia.
